# Interaction between dietary acrylamide intake and genetic variants for estrogen receptor-positive breast cancer risk

**DOI:** 10.1007/s00394-018-1619-z

**Published:** 2018-02-14

**Authors:** Janneke G. F. Hogervorst, Piet A. van den Brandt, Roger W. L. Godschalk, Frederik-Jan van Schooten, Leo J. Schouten

**Affiliations:** 1 Centre for Environmental Sciences, Hasselt University, Diepenbeek, Belgium; 2 Department of Epidemiology, School for Oncology and Developmental Biology (GROW), Maastricht University, Maastricht, The Netherlands; 3 Department of Pharmacology and Toxicology, School for Nutrition and Translational Research in Metabolism (NUTRIM), Maastricht University, Maastricht, The Netherlands

**Keywords:** Dietary acrylamide, Single nucleotide polymorphism, Estrogen receptor-positive breast cancer, Prospective cohort

## Abstract

**Purpose:**

The association between dietary acrylamide intake and estrogen receptor-positive (ER+) breast cancer risk in epidemiological studies is inconsistent. By analyzing gene-acrylamide interactions for ER+ breast cancer risk, we aimed to clarify the role of acrylamide intake in ER+ breast cancer etiology.

**Methods:**

The prospective Netherlands Cohort Study on diet and cancer includes 62,573 women, aged 55–69 years. At baseline, a random subcohort of 2589 women was sampled from the total cohort for a case–cohort analysis approach. Dietary acrylamide intake of subcohort members (*n* = 1449) and ER+ breast cancer cases (*n* = 844) was assessed with a food frequency questionnaire. We genotyped single nucleotide polymorphisms (SNPs) in genes in acrylamide metabolism, sex steroid systems, oxidative stress and DNA repair. Multiplicative interaction between acrylamide intake and SNPs was assessed with Cox proportional hazards analysis, based on 20.3 years of follow-up.

**Results:**

Unexpectedly, there was a statistically non-significant inverse association between acrylamide and ER+ breast cancer risk among all women but with no clear dose–response relationship, and no association among never smokers. Among the results for 57 SNPs and 2 gene deletions, rs1056827 in *CYP1B1*, rs2959008 and rs7173655 in *CYP11A1*, the *GSTT1* gene deletion, and rs1052133 in *hOGG1* showed a statistically significant interaction with acrylamide intake for ER+ breast cancer risk.

**Conclusions:**

This study did not provide evidence for a positive association between acrylamide intake and ER+ breast cancer risk. If anything, acrylamide was associated with a decreased ER+ breast cancer risk. The interaction with SNPs in *CYP1B1* and *CYP11A1* suggests that acrylamide may influence ER+ breast cancer risk through sex hormone pathways.

**Electronic supplementary material:**

The online version of this article (10.1007/s00394-018-1619-z) contains supplementary material, which is available to authorized users.

## Introduction

Acrylamide, a probable human carcinogen (IARC class 2A), was discovered in 2002 in various heat-treated carbohydrate-rich foods, such as cookies, potato crisps, French fries and coffee. Acrylamide is a small hydrophilic compound that is distributed throughout the body with the blood. In theory, it can thus cause cancer everywhere in the body. Acrylamide is a multisite carcinogen in rodents, in which it causes, among other, mammary gland tumors in females [1]. The mechanisms by which acrylamide causes mammary gland tumors in rodents are hypothesized to be genotoxicity and endocrine effects [1]. Since 2002, a few epidemiological studies have investigated the impact of dietary acrylamide intake on human cancer risks. The results of these studies for breast cancer are inconsistent. A recent meta-analysis did not show an increased risk of breast cancer overall, with a relative risk in the highest category of intake versus the lowest of 0.96 (95% CI 0.91–1.02) [2]. However, our previous analysis in the Netherlands Cohort Study [3] and a Danish study using acrylamide hemoglobin adducts as a marker of internal acrylamide exposure [4] gave some indications for a positive association between acrylamide intake and estrogen receptor-positive (ER+) breast cancer risk. Nevertheless, the above-mentioned meta-analysis did not show an increased risk of this type of breast cancer associated with dietary acrylamide intake either [RR 0.98 (95% CI 0.89–1.08)] [2].

In the present study, we investigated whether genetic make-up modifies the association between acrylamide and ER+ breast cancer risk, thereby contributing to evidence on acrylamide’s possible mechanism of action and on the causality of the observed associations. We focused on ER+ breast cancer because of the hypothesized effect of acrylamide on sex hormones and the fact that two studies observed an increased acrylamide-associated risk with this subtype of breast cancer. For ER+ breast cancers, the involvement of sex hormones in their etiology is probably stronger than for estrogen receptor-negative breast cancers [5]. We selected SNPs in candidate genes in acrylamide metabolism and in mechanisms through which acrylamide is hypothesized to cause cancer: mechanisms involving sex hormones, oxidative stress, and DNA damage caused by glycidamide, acrylamide’s genotoxic metabolite [6]. Previously, we investigated acrylamide intake and gene interactions for endometrial and ovarian cancer risk, and we observed indications for interaction between acrylamide intake and SNPs in among other *cytochrome P450, family 2, subfamily E, polypeptide 1* (*CYP2E1)* and the deletions of the genes *glutathione s-transferase M1 and T1* (*GSTM1* and *GSTT1)* [7, 8].

## Subjects and methods

### Study cohort, cases and follow-up

The Netherlands Cohort Study on diet and cancer started in September 1986 with the inclusion of 62,573 women that were 55–69 years of age, all presumed to be post-menopausal. Data on dietary habits and other risk factors were collected by means of a self-administered questionnaire at baseline in 1986. In addition to the questionnaire, approximately 75% of the participants sent in toenail clippings, as requested.

Following the case–cohort approach, ER+ breast cancer cases were enumerated for the entire cohort, while the accumulated person-years for the entire cohort were estimated from a subcohort of 2589 women randomly sampled from the entire cohort at baseline. Since the start of the study, the subcohort has been followed up regularly for vital status information. Incident cancer cases in the total cohort have been detected by computerized record linkages to the Netherlands Cancer Registry, the Netherlands Pathology Registry and the causes of death registry. Further details on the design of the study and methods of follow-up are presented elsewhere [9–12].

**Fig. 1:**
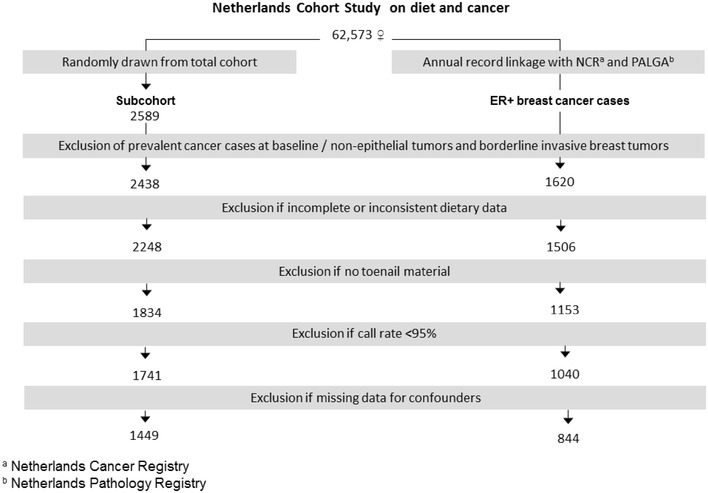
Flow chart of subcohort members and estrogen receptor-positive breast cancer cases

After 20.3 years of follow-up, from September 1986 to December 2006, and after exclusion of cohort members who reported a diagnosis of cancer (except skin cancer) at baseline, there were 1620 microscopically confirmed invasive ER+ primary carcinomas of the breast ([ICD-O]-3: C50). Information on estrogen receptor status was obtained from the National Cancer Registry and the Dutch Pathology Registry and was assessed by either immunohistochemistry or biochemical assay. Cases and subcohort members were excluded from analysis if their dietary data were incomplete or inconsistent, if they had not sent in toenail clippings, and if they had no or inferior (call rate < 95%) data on SNPs. Figure 1 shows the selection and exclusion steps that resulted in the numbers of cases and subcohort members that were available for analysis.

### Acrylamide intake assessment

A valid and reproducible food frequency questionnaire with questions on 150 food items was used for estimating dietary habits [11, 12]. Dietary acrylamide intake was estimated from the mean acrylamide level of foods on the Dutch market, and the frequency of consumption and portion size of the foods, as described in detail elsewhere [13].

### Selection of genes and SNPs

The selection of genes focused on genes involved in (1) acrylamide metabolism and (2) the most often hypothesized mechanisms of acrylamide-induced carcinogenesis [6]: (2a) sex hormonal effect (involving sex hormone synthesis/metabolism or sex hormone nuclear receptors), (2b) oxidative stress and (2c) genotoxicity (DNA repair), or (2d) SNPs in genes that have clearly been shown in the literature to play a role in carcinogenesis. A detailed description of the selection of genes and SNPs is presented elsewhere [7].

In the end, we genotyped 6 SNPs to determine *GSTM1 and GSTT1* deletions (3 SNPs each) and 60 SNPs in other genes, see Supplemental Table 1.

### DNA isolation and genotyping

DNA was isolated from 15 mg of toenail clippings, following the protocol developed by Cline et al. [14], in an optimised form [15]. Genotyping was performed by Agena in Hamburg, on the MassARRAY platform using the iPLEX TM assay [16] This method has been successfully used before to genotype DNA from toenails [7, 15, 17, 18].

Supplemental Table 2 shows the 60 SNPs with their location, call frequencies, and HWE *p* value. 3 out of the 60 SNPs that were genotyped had a call rate < 80% and were not included in the analyses. 6 SNPs out of the remaining 57 SNPs did not adhere to Hardy–Weinberg equilibrium (HWE) (*p* < 0.05). With regard to the SNPs selected to represent the *GSTM1* deletion, rs10857795 was not called in 36%, rs200184852 in 42% and rs74837985 in only 2% of the subcohort. The latter value appears to be due to genotyping error. Therefore, we decided to base the assessment of the absence/presence of the *GSTM1* gene only on rs10857795 and rs200184852. 31% of the subcohort had a missing value for both rs10857795 and rs200184852. With regard to *GSTT1*, rs2844008 was not called in 58%, rs4630 in 16%, and rs140309 in 11% of the subcohort. 8% of the subcohort had a missing value for all 3 *GSTT1* SNPs.

5% of the samples (*n =* 190) were duplicate samples in order to check the reproducibility of genotyping, which was > 99%. We excluded samples with a call rate < 95% (113 breast cancer cases, 93 subcohort members).

### Statistical analysis

Hazard rate ratios (HRs) and 95% confidence intervals were obtained through Cox proportional hazards regression with STATA software (package 13), using the robust Huber–White sandwich estimator to account for additional variance introduced by sampling from the cohort. We checked the proportional hazards assumption using scaled Schoenfeld residuals.

Covariables selected for inclusion in the Cox proportional hazards analysis models were selected based on the literature: age, body mass index, height, age at menarche, age at menopause, age at first childbirth, parity, ever use of oral contraceptives, ever use of postmenopausal hormones, history of benign breast disease, family history of breast cancer and energy intake. Smoking status, the duration of smoking and the number of cigarettes per day were included in the model, because cigarette smoke contains acrylamide. Smokers have on average four times higher levels of acrylamide-hemoglobin adducts than non-smokers [19, 20]. To eliminate the influence of acrylamide through smoking, we performed subgroup analyses restricted to never smokers. In addition, we checked the confounding potential of various dietary factors, e.g., alcohol, fibre, glycaemic index, but none changed the hazard ratio of acrylamide by more than 10%. The main associations between SNPs and ER+ breast cancer risk were only adjusted for age.

Multiplicative interaction between acrylamide intake and SNPs was tested using product terms of the continuous acrylamide intake variable and genotype. For statistical power reasons, we used a dominant genetic model for all SNPs. Tests for acrylamide dose–response trends in strata of the genotypes were performed by fitting the mean acrylamide intake in the tertiles as a continuous variable.

We applied the false discovery rate method developed by Benjamini–Hochberg to adjust for multiple testing [21] with the expected proportion of false positives set at 20% [22, 23].

Two-sided *p* values are reported throughout this paper.

## Results

Table 1 shows the characteristics of the subcohort and ER+ breast cancer cases at baseline. Cases reported to have fewer children and to more often be nulliparous than subcohort members. Cases reported more often to be current smokers, to have smoked more cigarettes and for a longer duration, and to have drunk more alcohol than subcohort members. They reported more often to have ever used postmenopausal hormone treatment and less often to have ever used oral contraceptives. Furthermore, cases reported more often to have a personal history of benign breast disease and family history of breast cancer.

**Table 1:** Characteristics of subcohort and estrogen receptor-positive breast cancer cases

Variable	ER+ breast cancer cases	Subcohort
*n*	364	1474
Dietary variables		
Acrylamide intake (µg/day)	20.6 (11.3)	21.0 (11.8)
Coffee (g/day)	498 (247)	499 (242)
Dutch spiced cake (g/day)	5.3 (9.0)	5.6 (9.5)
Cookies (g/day)	13.4 (11.5)	13.7 (10.6)
Potato crisps (g/day)	0.36 (1.36)	0.39 (1.80)
French fries (g/day)	3.8 (8.3)	3.7 (8.1)
Alcohol intake (g/day)	6.4 (10.7)	5.9 (9.6)
Total energy intake (kcal)	1689 (394)	1688 (396)
Non-dietary variables		
Age (years)	61.2 (4.2)	61.4 (4.3)
Height (cm)	166 (6)	165 (6)
BMI (kg/m^2^)	25.3 (3.3)	25.1 (3.6)
Age at menarche (years)	13.5 (1.8)	13.7 (1.8)
Age at menopause (years)	49.0 (4.5)	48.8 (4.4)
Parity, *n* children	2.5 (2.0)	2.8 (2.2)
Age at first childbirth		
Nulliparous	20.7	18.1
15–19 years	1.5	2.0
20–24 years	19.3	20.7
25–29 years	38.3	41.2
> 30 years	20.2	18.0
*n* cigarettes per day	5.0 (8.1)	4.5 (7.7)
*n* cigarette smoking years	12.0 (16.1)	11.2 (15.6)
Cigarette smoking status %		
Never smokers	56.4	58.8
Former smokers	22.0	21.2
Current smokers	21.6	20.0
Ever use of postmenopausal hormone treatment, % yes	14.5	13.6
Ever use of oral contraceptives, % yes	24.7	25.5
History of benign breast disease, % yes	12.3	7.3
Family history of breast cancer, % yes	14.1	8.4

*n* represents number of subcohort members or cases after exclusion of participants with prevalent cancer at baseline, incomplete or inconsistent dietary data, and a sample call rate < 95%. The number of missing values varies for the variables in this Table

There was a statistically non-significant inverse association between acrylamide and ER+ breast cancer risk after 20.3 years of follow-up in all women (HR of highest versus the lowest quintile of intake: 0.85 (95% CI 0.66–1.09) and 0.94 (0.88–1.00) per 10 µg/day increment of intake) but with no clear dose–response relationship. There was no association in never-smoking women (HR of highest versus the lowest quintile of intake: 1.18 (95% CI 0.85–1.64) and 1.02 (0.93–1.11) per 10 µg/day increment of intake) (Table 2).

**Table 2:** Main association between acrylamide intake and estrogen receptor-positive breast cancer risk, 20.3 years of follow-up

Main effect acrylamide	*N* cases	HR Per 10 µg/day increment	HR Quintile 1	HR Quintile 2	HR Quintile 3	HR Quintile 4	HR Quintile 5	*p* trend
All women	1238	0.94 (0.88–1.00)	Ref (1.00)	0.88 (0.69–1.11)	1.01 (0.79–1.29)	0.93 (0.73–1.20)	0.85 (0.66–1.09)	0.37
Never-smoking women	703	1.02 (0.93–1.11)	Ref (1.00)	1.08 (0.78–1.49)	1.44 (1.04–2.01)	1.34 (0.96–1.86)	1.18 (0.85–1.64)	0.17

Hazard ratios (HR) are adjusted for age (years), age at menarche (years), age at menopause (years), age at first childbirth (nulliparous, 15–19 years, 20–24 years, 25–29 years, ≥ 30 years), parity (*n* children), ever use of oral contraceptives (yes/no), ever use of postmenopausal hormone treatment (yes/no), height (cm), body mass index (kg/m^2^), educational level (4 levels) energy intake (kcal/day), history of benign breast disease, family history of breast cancer, and in the analyses for all women: smoking status (never/ex/current smoker), smoking quantity (*n* cigarettes/day), smoking duration (smoking years)

Table 3 presents the SNPs showing a trend for ER+ breast cancer over the number of variant alleles after 20.3 years of follow-up. None of the SNPs was statistically significantly associated with ER+ breast cancer risk after adjustment for multiple comparisons. There were some nominally statistically significant interactions. There was a statistically non-significant decrease in risk with an increasing number of variant alleles for rs2070959 in *UDP glucuronosyltransferase family 1 member A complex (UGT1A)* (*p* trend = 0.08), rs4919682 and rs4919687 in *cytochrome P450, family 17, subfamily A, polypeptide 1 (CYP17A1)* (*p* trend = 0.07 and 0.05, respectively), rs915906 in *CYP2E1* (*p* trend = 0.09), and rs3219489 in *human MutY homolog (hMYH)* (*p* trend = 0.07).

**Table 3:** Genetic variants showing a trend for estrogen receptor-positive breast cancer risk, 20.3 years of follow-up

Main effects SNPs	Total *N* cases	1 or 2 variant alleles versus homozygous wild type		1 variant allele versus homozygous wild type		2 variant alleles versus homozygous wild type		*p* trend per allele	Benjamini–Hochberg adjusted *p* value*
		*N* cases	HR (95% CI)^†^	*N* cases	HR (95% CI)^†^	*N* cases	HR (95% CI)^†^		
*UGT1A*, rs2070959	1040	536	0.87 (0.75–1.02)	450	0.88 (0.75–1.04)	86	0.83 (0.62–1.10)	0.08	0.84
*CYP17A1*, rs4919682	1039	477	0.82 (0.70–0.95)	392	0.79 (0.67–0.93)	85	0.95 (0.71–1.28)	0.07	0.84
*CYP17A1*, rs4919687	1040	494	0.82 (0.71–0.96)	407	0.81 (0.69–0.96)	87	0.89 (0.67–1.18)	0.05	0.84
*CYP2E1*, rs915906	1039	255	0.82 (0.69–0.98)	230	0.80 (0.66–0.96)	25	1.16 (0.68–1.98)	0.09	0.84
*hMYH*, rs3219489	1039	425	0.91 (0.78–1.06)	378	0.95 (0.81–1.12)	47	0.67 (0.47–0.97)	0.07	0.84

^†^Hazard ratios (HR) are adjusted for age

*Proportion of false positives threshold set at 0.2

Table 4 shows the interactions between SNPs and acrylamide intake that remained statistically significant after adjustment for multiple comparisons.

**Table 4:** Statistically significant interactions^a^ between SNPs and dietary acrylamide intake on the risk of estrogen receptor-positive breast cancer, 20.3 years of follow-up

SNP	Acrylamide, continuous intake	Acrylamide, tertiles of intake						Interaction	
	HR 10 µg/day	*N* cases	HR Tertile 1	*N* cases	HR Tertile 2	*N* cases	HR Tertile 3	*p* for linear interaction	
								Raw *p*	Benjamini–Hochberg adjusted *p* value^§^
All									
*CYP1B1*, rs1056827 = 0	0.87 (0.78–0.98)	150	Ref (1.00)	141	0.97 (0.71–1.35)	141	0.83 (0.60–1.15)	0.003	0.18
*CYP1B1*, rs1056827 = 1	1.05 (0.94–1.18)	134	Ref (1.00)	143	1.08 (0.77–1.50)	132	1.06 (0.75–1.49)		
Never smokers									
*CYP1B1*, rs1056827 = 0	0.95 (0.81–1.10)	84	Ref (1.00)	78	1.06 (0.68–1.66)	86	0.98 (0.64–1.51)	0.03	0.44
*CYP1B1*, rs1056827 = 1	1.16 (0.99–1.36)	68	Ref (1.00)	90	1.49 (0.97–2.30)	78	1.35 (0.87–2.10)		
All									
*CYP11A1*, rs2959008 = 0	0.83 (0.73–0.95)	133	Ref (1.00)	135	1.03 (0.71–1.50)	109	0.73 (0.51–1.06)	0.01	0.19
*CYP11A1*, rs2959008 = 1	1.04 (0.94–1.16)	153	Ref (1.00)	151	1.05 (0.77–1.42)	164	1.10 (0.81–1.50)		
Never smokers									
*CYP11A1*, rs2959008 = 0	0.87 (0.74–1.02)	73	Ref (1.00)	77	1.42 (0.86–2.35)	66	0.92 (0.56–1.49)	0.02	0.41
*CYP11A1*, rs2959008 = 1	1.18 (1.02–1.36)	80	Ref (1.00)	92	1.23 (0.82–1.86)	98	1.35 (0.90–2.03)		
All									
*CYP11A1*, rs7173655 = 0	0.84 (0.74–0.95)	139	Ref (1.00)	142	1.07 (0.76–1.51)	108	0.71 (0.50–1.02)	0.01	0.19
*CYP11A1*, rs7173655 = 1	1.03 (0.93–1.14)	146	Ref (1.00)	144	1.02 (0.75–1.40)	165	1.11 (0.81–1.452)		
Never smokers									
*CYP11A1*, rs7173655 = 0	0.89 (0.75–1.04)	74	Ref (1.00)	80	1.33 (0.83–2.14)	65	0.89 (0.55–1.43)	0.02	0.41
*CYP11A1*, rs7173655 = 1	1.17 (1.02–1.34)	78	Ref (1.00)	89	1.27 (0.83–1.93)	99	1.43 (0.94–2.17)		
All									
*GSTT1* present, rs140309	0.97 (0.90–1.06)	252	Ref (1.00)	251	1.02 (0.80–1.30)	255	1.00 (0.78–1.27)	0.01	0.19
*GSTT1* deleted, rs140309	0.66 (0.48–0.91)	34	Ref (1.00)	35	1.33 (0.55–3.21)	18	0.35 (0.15–0.83)		
Never smokers									
*GSTT1* present, rs140309	1.08 (0.97–1.21)	132	Ref (1.00)	148	1.26 (0.91–1.76)	152	1.29 (0.93–1.78)	0.01	0.41
*GSTT1* deleted, rs140309	0.61 (0.37–0.99)	21	Ref (1.00)	21	0.74 (0.21–2.68)	12	0.16 (0.04–0.68)		
All									
*hOGG1*, rs1052133 = 0	1.03 (0.94–1.14)	152	Ref (1.00)	161	1.08 (0.79–1.46)	182	1.19 (0.89–1.60)	0.02	0.19
*hOGG1*, rs1052133 = 1	0.81 (0.70–0.93)	134	Ref (1.00)	125	0.91 (0.63–1.31)	91	0.56 (0.37–0.83)		
Never smokers									
*hOGG1*, rs1052133 = 0	1.07 (0.95–1.21)	78	Ref (1.00)	92	1.39 (0.93–2.07)	112	1.36 (0.93–2.00)	0.26	0.90
*hOGG1*, rs1052133 = 1	0.95 (0.78–1.15)	75	Ref (1.00)	77	1.06 (0.63–1.76)	52	0.81 (0.48–1.38)		

Hazard ratios (HR) are adjusted for age (years), age at menarche (years), age at menopause (years), age at first childbirth (nulliparous, 15–19 years, 20–24 years, 25–29 years, ≥ 30 years), parity (*n* children), ever use of oral contraceptives (yes/no), ever use of postmenopausal hormone treatment (yes/no), height (cm), body mass index (kg/m^2^), educational level (4 levels) energy intake (kcal/day), history of benign breast disease, family history of breast cancer, and in the analyses for all women: smoking status (never/ex/current smoker), smoking quantity (*n* cigarettes/day), smoking duration (smoking years)

^§^Proportion of false positives threshold set at 0.2

^a^After adjustment for multiple comparisons

The homozygous deletion of *GSTT1*, when represented by rs140309, showed a statistically significant interaction with acrylamide intake (*p* interaction = 0.01) and this interaction remained statistically significant after adjustment for multiple testing (Benjamini–Hochberg adjusted *p* value 0.19). The same interaction was observed for never smokers. Women with a homozygous deletion of the *GSTT1* gene were at a statistically significantly decreased acrylamide-associated risk of ER+ breast cancer [HR 0.35 (95% CI 0.15–0.83) among all women and HR 0.16 (95% CI 0.04–0.68) among never-smoking women in the highest tertile of acrylamide intake versus the lowest], while there was no association with acrylamide intake among women with at least 1 copy of the gene.

There were four more interactions that remained statistically significant after adjustment for multiple testing, with the following SNPs: rs1056827 in *cytochrome P450, family 1, subfamily B, polypeptide 1 (CYP1B1)* (Benjamini–Hochberg adjusted *p* value 0.18), rs2959008 and rs7173655 (*R*^2^ 0.79, *D*′ 0.92) in *cytochrome P450, family 11, subfamily A, polypeptide 1* (*CYP11A1*) (Benjamini–Hochberg adjusted *p* value 0.19 for both SNPs), and rs1052133 in *human 8-oxo-7,8-dihydroguanine DNA glycosylase 1 (hOGG1)* (Benjamini–Hochberg adjusted *p* value 0.19). Women homozygous for the wild-type allele of *CYP1B1* were at a statistically significantly decreased acrylamide-associated risk of ER+ breast cancer, while women with at least 1 variant allele were not. Among never smokers, this pattern was not seen, although the interaction was nominally statistically significant (*p* interaction = 0.03). In this subgroup, there was a statistically non-significant increase in risk in women with at least one variant allele, while there was no association between acrylamide and risk in women with two wild-type alleles. The two SNPs in *CYP11A1* both showed that women homozygous for the wild-type allele were at a decreased acrylamide-associated risk while there was no association for women with at least one variant allele. This was seen both among all women and among never smokers. Only women with one or two variant alleles of rs1052133 in *hOGG1* were at a decreased acrylamide-associated risk of ER+ breast cancer. Women who were homozygous wild types did not show an association between acrylamide and ER+ breast cancer. The effect modification was less clear among never smokers.

In Table 5, we show interactions with SNPs in (other) genes involved in acrylamide metabolism that are interesting because they have a higher a priori probability of modifying the association between acrylamide and cancer risk than the other selected SNPs. Rs915906, rs2480258, and rs6413432 in *CYP2E1* did not show an interaction with acrylamide intake among all women, nor among never-smoking women. There was also no interaction between the deletion of *GSTM1* and acrylamide, or SNPs in other acrylamide-metabolizing genes.

**Table 5:** Interactions between SNPs in acrylamide-metabolizing genes and dietary acrylamide intake on the risk of estrogen receptor-positive breast cancer, 20.3 years of follow-up

SNP	Acrylamide, continuous intake	Acrylamide, tertiles of intake							Interaction	
	HR 10 µg/day	*N* cases	HR Tertile 1	*N* cases	HR Tertile 2	*N* cases	HR Tertile 3	*p* for trend	*p* for linear interaction	
									Raw *p*	Benjamini–Hochberg adjusted *p* value^§^
All										
*CYP2E1*, rs915906 = 0	0.93 (0.85–1.02)	212	Ref (1.00)	219	1.03 (0.79–1.35)	213	0.87 (0.67–1.14)	0.30	0.84	0.90
*CYP2E1*, rs915906 = 1	0.95 (0.79–1.15)	74	Ref (1.00)	67	1.03 (0.64–1.65)	59	0.96 (0.58–1.58)	0.86		
Never smokers										
*CYP2E1*, rs915906 = 0	1.02 (0.91–1.14)	111	Ref (1.00)	132	1.30 (0.91–1.85)	127	1.10 (0.77–1.55)	0.67	0.94	0.97
*CYP2E1*, rs915906 = 1	1.07 (0.82–1.39)	42	Ref (1.00)	37	1.19 (0.59–2.40)	37	1.24 (0.62–2.48)	0.55		
All										
*CYP2E1*, rs2480258 = =0	0.91 (0.82–1.01)	188	Ref (1.00)	185	0.99 (0.74–1.33)	181	0.83 (0.62–1.12)	0.21	0.48	0.83
*CYP2E1*, rs2480258 = 1	1.00 (0.87–1.14)	98	Ref (1.00)	101	1.09 (0.74–1.60)	92	1.02 (0.69–1.51)	0.93		
Never smokers										
*CYP2E1*, rs2480258 = 0	1.01 (0.89–1.14)	96	Ref (1.00)	110	1.29 (0.87–1.91)	105	1.12 (0.77–1.63)	0.60	0.68	0.95
*CYP2E1*, rs2480258 = 1	1.05 (0.86–1.28)	57	Ref (1.00)	59	1.11 (0.65–1.88)	59	1.06 (0.63–1.78)	0.83		
All										
*CYP2E1*, rs6413432 = 0	0.92 (0.84–1.00)	236	Ref (1.00)	229	1.02 (0.79–1.32)	225	0.85 (0.66–1.09)	0.19	0.27	0.70
*CYP2E1*, rs6413432 = 1	1.05 (0.85–1.30)	50	Ref (1.00)	57	1.04 (0.58–1.85)	48	1.13 (0.60–2.11)	0.70		
Never smokers										
*CYP2E1*,rs6413432 = 0	1.01 (0.91–1.13)	123	Ref (1.00)	136	1.33 (0.93–1.88)	133	1.13 (0.80–1.58)	0.53	0.40	0.90
*CYP2E1*, rs6413432 = 1	1.15 (0.80–1.66)	30	Ref (1.00)	33	0.87 (0.41–1.84)	31	1.10 (0.48–2.53)	0.81		
All										
*GSTM1* present, all SNPs	0.95 (0.86–1.05)	193	Ref (1.00)	198	1.05 (0.79–1.39)	179	0.86 (0.65–1.15)	0.29	0.82	0.90
*GSTM1* deleted, all SNPs	0.92 (0.79–1.06)	93	Ref (1.00)	88	1.02 (0.66–1.56)	94	1.06 (0.68–1.66)	0.79		
Never smokers										
*GSTM1* present, all SNPs	1.06 (0.94–1.20)	93	Ref (1.00)	111	1.55 (1.04–2.30)	107	1.26 (0.86–1.84)	0.27	0.22	0.86
*GSTM1* deleted, all SNPs	0.92 (0.74–1.13)	60	Ref (1.00)	58	1.00 (0.57–1.77)	57	1.00 (0.56–1.77)	1.00		
All										
*GSTP1*, rs1695 = 0	0.93 (0.82–1.06)	123	Ref (1.00)	116	0.86 (0.60–1.24)	115	0.80 (0.55–1.15)	0.22	0.82	0.90
*GSTP1*, rs1695 = 1	0.95 (0.86–1.05)	163	Ref (1.00)	170	1.16 (0.86–1.57)	158	0.98 (0.72–1.33)	0.88		
Never smokers										
*GSTP1*, rs1695 = 0	1.05 (0.87–1.26)	68	Ref (1.00)	74	1.23 (0.74–2.03)	70	1.00 (0.61–1.63)	0.96	0.74	0.95
*GSTP1*, rs1695 = 1	1.03 (0.90–1.16)	85	Ref (1.00)	95	1.33 (0.88–1.99)	94	1.22 (0.82–1.83)	0.35		
All										
*GSTA5*, rs4715354 = 0	0.99 (0.83–1.17)	91	Ref (1.00)	67	0.77 (0.49–1.21)	74	0.80 (0.51–1.26)	0.34	0.53	0.83
*GSTA5*, rs4715354 = 1	0.92 (0.84–1.01)	195	Ref (1.00)	217	1.14 (0.87–1.49)	198	0.95 (0.72–1.24)	0.65		
Never smokers										
*GSTA5*, rs4715354 = 0	1.04 (0.85–1.27)	46	Ref (1.00)	43	1.11 (0.59–2.10)	47	1.09 (0.61–1.94)	0.78	0.77	0.95
*GSTA5*, rs4715354 = 1	1.02 (0.90–1.15)	107	Ref (1.00)	126	1.26 (0.88–1.81)	117	1.10 (0.76–1.58)	0.65		
All										
*EPHX1*, rs1051740 = 0	0.96 (0.84–1.09)	152	Ref (1.00)	133	0.74 (0.53–1.03)	131	0.70 (0.49–0.98)	0.04	0.91	0.95
*EPHX1*, rs1051740 = 1	0.93 (0.84–1.03)	134	Ref (1.00)	153	1.36 (0.99–1.87)	142	1.15 (0.84–1.58)	0.41		
Never smokers										
*EPHX1*, rs1051740 = 0	1.01 (0.86–1.19)	82	Ref (1.00)	88	0.95 (0.61–1.47)	81	0.83 (0.53–1.30)	0.41	0.90	0.97
*EPHX1*, rs1051740 = 1	1.01 (0.88–1.17)	71	Ref (1.00)	81	1.48 (0.95–2.32)	83	1.32 (0.85–2.05)	0.22		

Hazard ratios (HR) are adjusted for age (years), age at menarche (years), age at menopause (years), age at first childbirth (nulliparous, 15–19 years, 20–24 years, 25–29 years, ≥ 30 years), parity (*n* children), ever use of oral contraceptives (yes/no), ever use of postmenopausal hormone treatment (yes/no), height (cm), body mass index (kg/m^2^), educational level (4 levels) energy intake (kcal/day), history of benign breast disease, family history of breast cancer, and in the analyses for all women: smoking status (never/ex/current smoker), smoking quantity (*n* cigarettes/day), smoking duration (smoking years)

^§^Proportion of false positives threshold set at 0.2

Supplemental Table 3 shows the nominally statistically significant interactions that did not withstand adjustment for multiple testing, namely: rs1800566 in *NQO1* and rs6838248 in *SLC7A11* specifically among never smokers.

Finally, there were some clear differences in the association with acrylamide between genotypes without a statistically significant interaction, namely for: rs2070959 in *UGT1A*, rs11252859 in *AKR1C1*, rs11252887 in *AKR1C2*, rs1280350 in *MGC12965*, rs1042157 and rs6839 in *SULT1A1*, rs737865 in *COMT*, rs10432782 in *SOD1*, rs4880 and rs5746136 in *SOD2*, rs1047303 in the *HSD3B1*/*B2* gene cluster, rs6259 in *SHBG*, rs6759180 in *RRM2*, and rs2228001 in *XPC* (Supplemental Table 3).

## Discussion

As far as we know, this is the first study to analyze acrylamide-gene interactions for breast cancer risk. We observed interactions between acrylamide intake and rs1056827 in *CYP1B1*, rs2959008 and rs7173655 in *CYP11A1*, the *GSTT1* gene deletion, and rs1052133 in *hOGG1*. These interactions remained statistically significant after adjustment for multiple testing.

Contrary to what we found in a previous analysis for ER+ breast cancer albeit not statistically significant [3], acrylamide intake was not positively associated with ER+ breast cancer risk among never smokers in the current analysis. In addition, when we restricted our analyses to 13.3 years of follow-up (as in the previous analysis), acrylamide intake was not positively associated with ER+ breast cancer risk. The explanation for this discrepancy is probably that different case sets were used in the analyses. In the previous analyses, cases were derived from the Dutch Pathology Registry and four regional Dutch cancer registries because only those 4 routinely recorded information on estrogen receptor status at that time. Cases for the current analysis originated from all nine regional Dutch cancer registries, the Dutch Pathology Registry and the causes of death registry, and so there were more cases in the present analysis. In the previous analysis, the percentage of cases with missing info on estrogen receptor status was quite high (57%) and we checked whether cases with known estrogen receptor status differed from cases with unknown receptor status with regard to tumor and other characteristics, such as BMI and age. This was not the case but it is still possible that selection of a specific subgroup of ER+ cases occurred and that the positive association between acrylamide intake and ER+ breast cancer risk was restricted to this group. Among all women in the current analysis, there was a tendency towards an inverse association.

Glycidamide (the epoxide metabolite of acrylamide formed through metabolism by CYP2E1) is often thought to be responsible for acrylamide-induced carcinogenesis due to genotoxicity (mutagenicity and/or clastogenicity [24]). Studying the modifying effect of SNPs in *CYP2E1* on the association between acrylamide and cancer risk may thus contribute important information on the causality of the association. We observed no interaction between 3 SNPS in *CYP2E1* and acrylamide intake for ER+ breast cancer risk and there were no clear differences in the risk between the genotypes, contrary to what was observed for endometrial [7] and ovarian cancer [8]. We have no clear explanation for this inconsistency but an explanation could be that there is no association between acrylamide intake and breast cancer risk or that for breast cancer, acrylamide and glycidamide are (roughly) equally responsible for the carcinogenic effect. The three studied *CYP2E1* SNPs are in the intronic region of the gene and there is no clear information about their functionality but the wild-type allele of rs6413432 has been shown to be associated with an increased risk of prostate cancer [25] and other cancers such as lung cancer [26].

We observed that women with a homozygous deletion of *GSTT1* (deletion represented by rs140309) were at a decreased acrylamide-associated risk of ER+ breast cancer but the number of cases with a homozygous *GSTT1* deletion was rather small (*n =* 87). In contrast, we previously observed women with at least one copy of the *GSTT1* gene to be at an increased acrylamide-associated risk of endometrial and ovarian cancer [7, 8]. There was no clear difference in the association between acrylamide intake and ER+ breast cancer risk between the genotypes of *GSTM1*.

We observed a statistically significant interaction between acrylamide intake and rs1056827 in *CYP1B1*. Women who were homozygous wild types for this allele were at a decreased risk of acrylamide-associated ER+ breast cancer. In never smokers, the interaction was less clear. CYP1B1 is a phase I biotransformation enzyme involved in the metabolism of various exogenous and endogenous compounds and is mainly expressed in endocrine tissues like the endometrium, ovarium and breast. CYP1B1 converts estrogens to hydroxy metabolites (catechol estrogens) which are potent estrogens and furthermore CYP1B1 oxidizes catechol estrogens to chemically reactive semiquinone and quinone intermediates that can bind to DNA and cause mutations. Exposure of mouse spermatocytes to acrylamide and glycidamide led to increased *CYP1B1* expression [27], while glycidamide exposure led to decreased *CYP1B1* expression in human epithelial cells [28]. Possible explanations for this discrepancy are species, cell or dose differences. The variant allele of rs1056827 has been shown to have increased enzyme activity and to be associated with an increased breast cancer risk [29]. Due to the scarcity of literature and the inconsistency in the relationship between acrylamide and CYP1B1 activity, it is currently impossible to say whether the observed interaction is biologically plausible.

Acrylamide interacted statistically significantly with 2 SNPs in *CYP11A1*: rs2959008 and rs7173655; acrylamide intake was associated with a decreased ER+ breast cancer risk in women who were homozygous for the wild type of these alleles. CYP11A1 is involved in the formation of pregnenolone from cholesterol, the first and rate-limiting step in steroid hormone synthesis. Both *CYP11A1* SNPs are in the intronic region of the gene and there is no information available about their functionality. The variant allele of rs2959008 was associated with a decreased breast cancer risk in Han Chinese [30]. There is no literature on rs7173655 and breast cancer risk but the variant allele was associated with an increased endometrial cancer risk [31]. Acrylamide exposure of male Fischer 344 rats led to increased expression of *CYP11A1* in reproductive tissues [32] but to decreased *CYP11A1* expression in testis tissue of male Sprague–Dawley rats [33]. This discrepancy may be due to differences in rat strains or doses of acrylamide, or both. Thus, again due to the scarcity and inconsistency of data, it is not currently possible to judge the biological plausibility of the interaction between acrylamide and these *CYP11A1* SNPs.

Progesterone opposes the proliferative effect of estrogens in the endometrium and ovaries while it is thought to have proliferative effects in mammary tissue [34]. A mechanism by which acrylamide may increase risks of endometrial and ovarian cancer and decrease the risk of ER+ breast cancer is through an effect on progesterone. However, this is highly speculative, also due to the fact that we did not see interaction between acrylamide and the *CYP2E1* SNPs which leaves the possibility that there may not be a true association between acrylamide intake and breast cancer risk. If true, the interactions between acrylamide and *HSD3B* SNPs for ovarian cancer [8] and between acrylamide and the *CYP1B1* and *CYP11A1* SNPs for breast cancer in the current study give some indications that acrylamide may interfere with progesterone metabolism. However, there was no association between acrylamide intake and progesterone in a cross-sectional study in premenopausal women [35]. In animals, acrylamide has repeatedly been shown to decrease progesterone levels [36–38]. Nevertheless, we strongly encourage more research on the possible effect of acrylamide on progesterone metabolism in humans.

Only women with variant alleles of rs1052133 in *hOGG1* showed an inverse association between acrylamide intake and ER+ breast cancer risk. The *hOGG1* gene is part of the base excision DNA repair pathway, responsible for the excision of 8-oxoguanine (8-oxoG), a mutagenic DNA base byproduct of reactive oxygen species. Although the variant allele is hypothesized to have decreased enzyme activity [39, 40], a recent meta-analysis showed that it is associated with a decreased risk of breast cancer among Europeans [41], while another meta-analysis did not show an association [42]. Because of these apparent contradictions, it is currently impossible to speculate about the possible mechanism by which this SNP could modify the association between acrylamide intake and ER+ breast cancer risk.

There were two other nominally statistically significant interactions between acrylamide intake and other SNPs: rs1800566 in *NQO1* and rs6838248 in *SLC7A11*. Additionally, there were some clear differences in the association with acrylamide between genotypes without a statistically significant interaction, for: rs2070959 in *UGT1A*, rs11252859 in *AKR1C1*, rs11252887 in *AKR1C2*, rs1280350 in *MGC12965*, rs1042157 and rs6839 in *SULT1A1*, rs737865 in *COMT*, rs10432782 in *SOD1*, rs4880 and rs5746136 in *SOD2*, rs1047303 in the *HSD3B1*/*B2* gene cluster, rs6259 in *SHBG*, rs6759180 in *RRM2*, and rs2228001 in *XPC*. For all these SNPs it is even more important that the interaction between acrylamide intake and these SNPs is corroborated in other studies to be able to judge whether our findings represent true interactions or not.

This study has some limitations. Acrylamide levels vary considerably within foods due to processing and varieties used. Despite the large variation in acrylamide levels within foods, acrylamide intake as assessed by food frequency questionnaires and acrylamide to hemoglobin adducts (biomarker for exposure) have been shown to correlate moderately in several studies, e.g. [43, 44]. Thus, the food frequency questionnaire is able to estimate the rank order of acrylamide intake among study populations. In addition, we correlated the assessed acrylamide intake (based on mean acrylamide levels per food) and the measured acrylamide content of 24-hour Dutch duplicate diets, for which the participants had written down exactly what and how much they ate and drank. The correlation was very high (*r* = 0.82, *p* < 0.001) [45]. To conclude, assessing acrylamide intake through food frequency questionnaires is not perfect and entails some random measurement error, which pushes the point estimate towards the null, but it is useful for studying the link between acrylamide intake and cancer risk. Data on diet and covariables obtained from the questionnaire were collected only once, at baseline. Some of the characteristics (e.g., diet, BMI) will certainly have changed over time after baseline. One of the reasons to select an elderly population for the study was that older people tend to have more stable dietary habits. The changes that have occurred despite this will have resulted in random measurement error, pushing the point estimate of the hazard ratio towards the null.

Some of the nominally statistically significant interactions that we observed are, without a doubt, chance findings. However, it is of interest that some of the genes that we observed to interact with acrylamide for ER+ breast cancer risk or that showed clear differences in risk between the genotypes also did so for endometrial [7] and ovarian cancer [8]: *GSTT1, AKR1C1, NQO1*, the *HSD3B1*/*B2* gene cluster, *XPC*, and *MGC12965*. These genes therefore deserve attention in future studies.

The strengths of this study are the complete follow-up and its prospective nature.

In conclusion, we did not observe a positive association between dietary acrylamide intake and ER+ breast cancer risk. Unexpectedly, our results gave some indications for an inverse association. Unlike for endometrial and ovarian cancer, there was no interaction between acrylamide intake and *CYP2E1* SNPs for ER+ breast cancer risk. After adjustment for multiple testing, this study showed statistically significant interactions between rs1056827 in *CYP1B1*, rs2959008 and rs7173655 in *CYP11A1*, the deletion of *GSTT1*, and rs1052133 in *hOGG1* and acrylamide intake for ER+ breast cancer risk. Based on this study and analyses for endometrial and ovarian cancer, we recommend follow-up of interactions between acrylamide intake and genetic polymorphisms in *CYP1B1, CYP11A1*, the *HSD3B1*/*B2* gene cluster, *CYP2E1, GSTs, hOGG1, AKR1C1, NQO1, GPX1, XPC* and *MGC12965*, and additional research on the possible effect of acrylamide on progesterone metabolism in humans.

## Electronic supplementary material

Below is the link to the electronic supplementary material.

Supplementary material 1 (DOC 1306 KB)
