# Supplementary material for: Interaction between dietary acrylamide intake and genetic variants for estrogen receptor-positive breast cancer risk
Source: Eur J Nutr. 2018 Feb 14;58(3):1033–45. doi: 10.1007/s00394-018-1619-z (PMC6499753; doi:10.1007/s00394-018-1619-z)
Supplement: Supplementary file 1 — Supplementary material 1 (DOC 1306 KB) [file 394_2018_1619_MOESM1_ESM.doc]

**SUPPLEMENTAL TABLES**

**Interaction between dietary acrylamide intake and genetic variants for estrogen receptor-positive breast cancer risk**

Janneke GF Hogervorst^1,2^, Piet A van den Brandt^2^, Roger WL Godschalk^3^, Frederik-Jan van Schooten^3^, Leo J Schouten^2^

^1^ Centre for Environmental Sciences, Hasselt University, Diepenbeek, Belgium.

^2^ Department of Epidemiology, School for Oncology & Developmental Biology (GROW), Maastricht University, Maastricht, the Netherlands

^3^ Department of Pharmacology and Toxicology, School for Nutrition and Translational Research in Metabolism (NUTRIM), Maastricht University, Maastricht, the Netherlands

email: [jgf.hogervorst@maastrichtuniversity.nl](mailto:jgf.hogervorst@maastrichtuniversity.nl);

Supplemental Table 1: Potentially interesting SNPs for investigating acrylamide-gene interactions (* eventually genotyped)

| **Gene** | **Reason for (non)selection (apart from association with cancer)** | | | **db SNP ID** | **MAF** | | | **Localization in gene and predicted functionality** | **Literature on association with cancer** |
| --- | --- | --- | --- | --- | --- | --- | --- | --- | --- |
| **Category 1: Acrylamide metabolism** | | | | | | | | | |
| ***CYP2E1*** | Polymorphism study | | |  | |  | |  |  |
|  |  | | | rs2480258* | | 25 | | Intronic, no info in F-SNP | No leads in HugeNavigator and PubMed (in Dec 2013) |
|  |  | | | rs915906* | | 17 | | Intronic, no info in F-SNP | No leads in HugeNavigator and PubMed (in Dec 2013) |
|  | *Not selected: MAF too low* | | | rs2031920 | | 6 | | Regulatory region, upstream, 0.40 | Associated with prostate cancer risk and breast cancer risk |
|  |  | | | rs6413432* | | 14 | | Intronic, no info in F-SNP | Associated with prostate cancer risk |
|  | *Not selected: MAF too low* | | | rs3813867 | | 7 | | Upstream, no info in F-SNP | No leads in HugeNavigator and PubMed (in Dec 2013) |
| ***EPHX1*** | Gene expression study  + polymorphism studies | | |  | |  | |  |  |
|  | Polymorphism study | | | rs1051740* | | 33 | | NS coding, 0.70 | Associated with ovarian cancer risk + breast cancer risk + prostate cancer risk |
|  | Polymorphism studies, | | | rs2234922 | | 21 | | NS coding, 0.14 | No leads in HugeNavigator and PubMed (in Dec 2013) |
| ***GSTA2*** | Polymorphism study  + gene expression study | | |  | |  | |  |  |
|  | *Not selected: MAF too low* | | | rs6577 | | 7 | | NS coding, 0.90 | No leads in HugeNavigator and PubMed (in Dec 2013) |
| ***GSTA5*** | Gene expression study | | |  | |  | |  |  |
|  | Highest F-value, sufficient MAF | | | rs4715354* | | 49 | | Intronic, 0.5 | No leads in HugeNavigator and PubMed(in Dec 2013) |
| ***GSTM1*** | Polymorphism studies  + gene expression study | | |  | |  | |  |  |
|  | Polymorphism studies | | | Gene deletion | | 50 | |  | Associated with breast cancer risk + prostate cancer risk + ovarian cancer risk + endometrial cancer risk |
|  | Selected to represent gene deletion | | | rs200184852* | | 22 | | NS coding, no info in F-SNP | No leads in HugeNavigator and PubMed(in Dec 2013) |
|  | Selected to represent gene deletion | | | rs74837985* | | 42 | | NS coding, no info in F-SNP | No leads in HugeNavigator and PubMed (in Dec 2013) |
|  | Selected to represent gene deletion | | | rs10857795* | | 10 | | Intronic, 0.24 | No leads in HugeNavigator and PubMed(in Dec 2013) |
| ***GSTM4*** | Gene expression study | | |  | |  | |  |  |
|  |  | | | rs560018 | | 37 | | Intronic, no info in F-SNP | No leads in HugeNavigator and PubMed(in Dec 2013) |
| ***GSTP1*** | Polymorphism study  + gene expression study | | |  | |  | |  |  |
|  | Polymorphism study | | | rs1695* | | 39 | | NS coding, 0.37 | Associated with breast cancer risk+ prostate cancer risk + endometrial cancer risk + ovarian cancer |
| ***GSTT1*** | Polymorphism studies, | | |  | |  | |  |  |
|  | Polymorphism studies | | | Gene deletion | | 18 | |  | Associated with prostate cancer risk + breast cancer risk + endometrial cancer risk |
|  | Selected to represent gene deletion | | | rs4630* | | 11 | | 3’-UTR, 0 | No leads in HugeNavigator and PubMed (in Dec 2013) |
|  | Selected to represent gene deletion | | | rs140309* | | 11 | | 3’-UTR, 0.5 | No leads in HugeNavigator and PubMed (in Dec 2013) |
|  | Selected to represent gene deletion | | | rs2844008* | | 11 | | Intron, 0.24 | No leads in HugeNavigator and PubMed (in Dec 2013) |
| ***Category 2a: Sex steroid biosynthesis, metabolism and receptors*** | | | | | | | | | |
| ***AKR1C1*** | | Gene expression study | |  | | |  |  |  |
|  | |  | | rs11252859* | | | 38 | Downstream, 0.66 | No leads in HugeNavigator and PubMed (in Dec 2013) |
| ***AKR1C2*** | | Gene expression study | |  | | |  |  |  |
|  | |  | | rs11252887* | | | 32 | Intergenic, 0.5 | Associated with endometrial cancer risk |
| ***AKR1C3*** | | Gene expression study | |  | | |  |  |  |
|  | |  | | rs12529 | | | 40 | NS coding, 0.5 | Associated with prostate cancer prognosis |
|  | |  | | rs7741* | | | 35 | S coding, conserved | Associated with hereditary prostate cancer risk |
|  | |  | | rs12387 | | | 16 | S coding | Associated with breast cancer risk among PHT users |
|  | |  | | rs4881400 | | | 24 | Intronic, no info in F-SNP | Associated with hereditary prostate cancer risk |
| ***COMT*** | |  | |  | | |  |  |  |
|  | |  | | rs4680* | | | 48 | NS coding, 0.86 | Associated with breast cancer risk meta-analysis + endometrial cancer risk |
|  | |  | | rs737865 | | | 32 | Intronic, 0.18 | Associated with breast cancer risk |
| ***CYP1A1*** | | Gene expression study | |  | | |  |  |  |
|  | |  | | rs4646903* | | | 10 | Downstream, no info in F-SNP | Associated with breast cancer risk + endometrial cancer risk + meta-analysis prostate cancer risk |
|  | | *Not selected: MAF too low* | | rs1048943 | | | 3 | NS coding, 0.13 | Associated with ovarian cancer risk meta-analysis + breast cancer risk + prostate cancer risk |
| ***CYP1A2*** | | Gene expression study | |  | | |  |  |  |
|  | |  | | rs762551* | | | 28 | Intronic, no info in F-SNP | Associated with breast cancer risk + ovarian cancer risk + risk of female cancers |
| ***CYP1B1*** | | Gene expression study | |  | | |  |  |  |
|  | |  | | rs1800440 | | | 19 | NS coding, 0.90 | Associated with endometrial cancer risk |
|  | |  | | rs1056827* | | | 36 | NS coding, 0.75 | Associated with breast cancer risk + prostate cancer risk |
|  | |  | | rs1056836* | | | 44 | NS coding, 0.86 | Associated with ovarian cancer risk + endometrial cancer risk + increased prostate cancer risk |
| ***CYP2C19*** | |  | |  | | |  |  |  |
|  | |  | | rs4917623 | | | 49 | Intronic, 0.21 | Associated with breast cancer risk |
| ***CYP3A4*** | |  | |  | | |  |  |  |
|  | | *Not selected: MAF too low* | | rs10273424 | | | 9 | Intergenic, no info in F-SNP | Associated with breast cancer risk + prostate cancer risk |
| ***CYP11A1*** | | Gene expression studies | |  | | |  |  |  |
|  | |  | | rs7173655* | | | 33 | Intronic, no info in F-SNP | Associated with endometrial cancer risk |
|  | |  | | rs4555110 | | | 16 | Intergenic, no info in F-SNP | Associated with endometrial cancer risk |
|  | |  | | rs3825944* | | | 17 | Intergenic, 0.77 | Associated with endometrial cancer risk |
|  | |  | | rs2959008* | | | 32 | Intronic, no info in F-SNP | Associated with breast cancer risk |
| ***CYP17A1*** | | Gene expression study | |  | | |  |  |  |
|  | |  | | rs743572* | | | 38 | 5 prime UTR, 0.05 | Associated with breast cancer risk + endometrial cancer risk + prostate cancer risk |
|  | |  | | rs4919682* | | | 28 | Intergenic, 0.5 | Marginally associated increased breast cancer risk |
|  | |  | | rs4919687* | | | 33 | Intronic, 0 | Marginally associated increased breast cancer risk |
| ***CYP19A1*** | |  | |  | | |  |  |  |
|  | |  | | rs4775936 | | | 47 | Intronic, 0 | Associated with endometrial cancer risk |
|  | |  | | rs727479 | | | 32 | Intronic, 0.18 | Associated with endometrial cancer risk + ovarian cancer risk |
|  | |  | | rs749292* | | | 46 | Intronic, 0.21 | Associated with increased endometrial cancer risk + increased ovarian cancer risk |
| ***HSD3B1/HSD3B2 cluster*** | | Gene expression study | |  | | |  |  |  |
|  | |  | | rs6203 | | | 39 | S coding, 0.33 | Associated with prostate cancer risk |
|  | |  | | rs1047303* | | | 35 | NS coding, 0.92 | Associated with prostate cancer risk |
|  | |  | | rs6428830 | | | 33 | Intronic, 0.10 | Associated with prostate cancer risk |
|  | |  | | rs4659175* | | | 32 | 5’ near gene, 0.21 | No leads in HugeNavigator and PubMed (in Dec 2013) |
|  | |  | | rs1538989* | | | 48 | Downstream, 0.10 | Associated with prostate cancer risk |
| ***HSD17B1*** | |  | |  | | |  |  |  |
|  | |  | | rs676387 | | | 34 | Intronic, 0.24 | Associated with breast cancer risk |
|  | |  | | rs605059 | | | 43 | NS coding, 0.20 | Associated with breast cancer Yao 2010 + prostate cancer risk |
| ***HSD17B3*** | | Gene expression study | |  | | |  |  |  |
|  | |  | | rs2257157* | | | 45 | Intronic, 0.21 | Associated with prostate cancer progression |
|  | |  | | rs2253502* | | | 20 | Intronic, 0.05 | Associated with prostate cancer risk |
| ***SHBG*** | |  | |  | | |  |  |  |
|  | |  | | rs6259* | | | 14 | NS coding, 0.5 | Associated with prostate cancer risk |
|  | |  | | rs1799941 | | | 23 | Intronic, no info in F-SNP | Associated with ovarian cancer risk |
| ***SRD5A1*** | | Gene expression studies | |  | | |  |  |  |
|  | |  | | rs10076470 | | | 38 | Splice site, intronic, 1.0 | No leads in HugeNavigator and PubMed (in Dec 2013) |
|  | |  | | rs3736316 | | | 38 | S coding, 0.11 | Modifies relationship between postmenopausal hormone treatment and postmenopausal breast cancer risk |
|  | |  | | rs8192120* | | | 36 | Intronic, 0.27 | Associated with endometrial cancer risk |
|  | |  | | rs824811* | | | 23 | Intronic, 0.22 | Associated with endometrial cancer risk |
| ***SRD5A2*** | |  | |  | | |  |  |  |
|  | |  | | rs523349 | | | 19 | NS coding, 0.33 | Associated with prostate cancer risk + prostate cancer risk meta-analysis |
|  | |  | | rs623419 | | | 43 | Intergenic, 0 | Associated with prostate cancer risk |
| ***SULT1A1*** | | Gene expression study | |  | | |  |  |  |
|  | | *Not selected: not possible to design primer* | | rs9282861 | | | 26 | NS coding, 0.75 | Associated with ovarian cancer risk + risk of female cancers+ modifies association between endogenous hormones and BMI and breast cancer risk |
|  | |  | | rs6839* | | | 28 | 3 prime UTR, no info in F-SNP | Associated with endometrial cancer risk |
|  | |  | | rs1042157* | | | 34 | 3 prime UTR, no info in F-SNP | Associated with endometrial cancer risk |
| ***SULT1E1*** | | Gene expression study | |  | | |  |  |  |
|  | |  | | rs3736599* | | | 14 | 5 prime UTR, 0.5 | Associated with endometrial cancer risk |
| ***UGT1A4/UGT1A9/UGT1A10*** | | Gene expression studies | |  | | |  |  |  |
| ***UGT1A6-10*** | |  | | rs2070959* | | | 31 | NS coding, 0.25 | Associated with endometrial cancer risk |
|  | |  | | rs10176426 | | | 12 | Intronic, 0.10 | Interaction with heterocyclic amines for prostate cancer risk |
|  | |  | | rs4663335 | | | 16 | Intergenic, no info in F-SNP | Interaction with heterocyclic amines for prostate cancer risk |
|  | |  | | rs6742078 | | | 31 | Intronic, 0.5 | Interaction with heterocyclic amines for prostate cancer risk |
| ***UGT2B17*** | | Gene expression study | |  | | |  |  |  |
|  | | *Not selected: takes 3 SNPs to genotype* | | Gene deletion | | | 12 |  | Associated with prostate cancer risk + breast cancer risk |
| ***ESR1*** | |  | |  | | |  |  |  |
|  | |  | | rs2077647 | | | 41 | S coding, 0.5 | Associated with breast cancer risk + endometrial cancer risk |
|  | |  | | rs2234693* | | | 41 | Intronic, 0.21 | Associated with endometrial cancer risk + prostate cancer risk |
|  | |  | | rs3798577 | | | 48 | 3 prime UTR, 0 | Increased breast cancer risk |
|  | |  | | rs9340799 | | | 31 | Intronic, 0.18 | Associated with breast cancer risk + endometrial cancer risk + prostate cancer risk meta-analysis |
|  | |  | | rs3020314 | | | 26 | Intronic, 0 | Associated with breast cancer risk + endometrial cancer risk meta-analysis |
| ***ESR2*** | |  | |  | | |  |  |  |
|  | |  | | rs4986938* | | | 38 | Intronic, 0 | Associated with breast risk + prostate cancer risk |
|  | |  | | rs1256030 | | | 42 | Intronic, 0.18 | Associated with ovarian cancer |
|  | |  | | rs2987983* | | | 31 | Intronic, 0.5 | Associated with breast cancer risk + prostate cancer risk |
| ***PGR*** | |  | |  | | |  |  |  |
|  | | *Not selected: not possible to design primer* | | rs3740753 | | | 26 | NS coding, 0.5 | Associated with ovarian cancer risk |
|  | |  | | rs660149* | | | 25 | Intronic, 0.18 | Associated with breast cancer risk |
|  | |  | | rs471767 | | | 31 | Downstream, 0.5 | Associated with endometrial cancer risk |
|  | |  | | rs1042838 | | | 20 | NS coding, 0.56 | Associated with ovarian cancer risk, + breast cancer risk + endometrial cancer risk |
| ***AR*** | |  | |  | | |  |  |  |
|  | |  | | rs6152 | | | 18 | S coding, 0.33 | Associated with prostate cancer risk + metastatic prostate cancer risk + endometrial cancer risk |
|  | |  | | rs7061037 | | | 19 | Intronic, 0.21 | Associated with prostate cancer risk |
|  | |  | | rs5964607 | | | 21 | Intergenic, no info in F-SNP | Associated with prostate cancer risk |
|  | |  | | rs5919393 | | | 17 | Intronic, 0.05 | Associated with endometrial cancer risk |
| ***Category 2b: Oxidative stress*** | | | | | | | | | |
| ***CAT*** | Gene expression study | | |  |  | | |  |  |
|  |  | | | rs4756146 | 8 | | | Intronic, 0.18 | Associated with postmenopausal breast cancer risk |
|  |  | | | rs1001179* | 25 | | | 5’ near gene, 0.24 | Associated with prostate cancer risk + interaction with fruits + vegetables for breast cancer risk + interaction with postmenopausal hormone treatment |
|  |  | | | rs511895* | 41 | | | Intronic, 0.21 | Associated with breast cancer risk |
|  |  | | | rs554518 | 15 | | | Upstream, no info in F-SNP | Associated with prostate cancer risk (borderline sign.) |
| ***GPX1*** | Gene expression study | | |  |  | | |  |  |
|  |  | | | rs1050450 | 19 | | | NS coding, 0.54 | Associated with breast cancer risk + prostate cancer risk + interaction with selenium for prostate cancer risk |
|  |  | | | rs3448* | 27 | | | 3-UTR, 0.30 | Associated with prostate cancer risk |
| ***CuZnSOD (SOD1)*** | Gene expression studies, | | |  |  | | |  |  |
|  |  | | | rs10432782* | 12 | | | Intronic, 0.21 | Interaction with selenium for prostate cancer risk |
| ***MnSOD (SOD2)*** | Gene expression study | | |  |  | | |  |  |
|  |  | | | rs4880* | 45 | | | NS coding, 0.33 | Associated with breast cancer risk + prostate cancer risk + ovarian cancer risk |
|  |  | | | rs5746136* | 33 | | | UTR-3, 0.18 | Associated with prostate cancer risk |
|  |  | | | rs2758330 | 25 | | | Intronic, 0 | Interaction with selenium for prostate cancer risk |
| ***TXN*** | Gene expression studies | | |  |  | | |  |  |
|  |  | | | rs2301241* | 41 | | | Upstream, no info in F-SNP | Associated with breast cancer risk |
| ***NQO1*** | Gene expression studies | | |  |  | | |  |  |
|  |  | | | rs1800566* | 22 | | | NS coding, 1 | Associated with breast cancer risk meta-analysis + prostate cancer risk + modifies association between oral contraceptives and breast cancer risk |
| ***Category 2c: DNA repair*** | | | | | | | | | |
| ***OGG1*** | Acrylamide-Hb adducts associated with urinary 8-OHdG adducts in a cross-sectional study in humans. OGG1 is a base excision repair gene that removes these adducts. | | |  |  | | |  |  |
|  |  | | | rs1052133* | 22 | | | NS coding, 0.29 | Associated with breast cancer risk meta-analysis + endometrial cancer risk + prostate cancer risk meta-analysis |
| ***XRCC1*** | Polymorphism study | | |  |  | | |  |  |
|  | Polymorphism study | | | rs25487* | 37 | | | NS coding, 0.41 | Associated with breast cancer risk + prostate cancer risk + endometrial cancer risk |
| ***PCNA*** | Gene expression study | | |  |  | | |  |  |
|  |  | | | rs3626 | 12 | | | 3-UTR, no info in F-SNP | No leads in HugeNavigator and PubMed (in Dec 2013) |
|  |  | | | rs25406 | 40 | | | Intronic, no info in F-SNP | No leads in HugeNavigator and PubMed (in Dec 2013) |
| ***MUTYH*** | Polymorphism study | | |  |  | | |  |  |
|  | Polymorphism study | | | rs3219489* | 28 | | | NS coding, 0.59 | No leads in HugeNavigator and PubMed (in Dec 2013) |
| ***XPC*** | Polymorphism study | | |  |  | | |  |  |
|  | Polymorphism study | | | rs2228000* | 29 | | | NS coding, 0.5 | Associated with breast cancer risk meta-analysis + endometrial cancer |
|  | Polymorphism study | | | rs2228001* | 41 | | | NS coding, 0.88 | Associated with endometrial cancer |
| ***Category 2d: Other*** | | | | | | | | | |
| ***CYP7B1*** | | | Gene expression study |  |  | | |  |  |
|  | | |  | rs7842714 | 47 | | | Intronic, 0.10 | No leads in HugeNavigator and PubMed (in Dec 2013) |
|  | | |  | rs656506 | 46 | | | Upstream, 0.5 | No leads in HugeNavigator and PubMed (in Dec 2013) |
| ***STAR*** | | | Gene expression study |  |  | | |  |  |
|  | | |  | rs6474491 | 19 | | | Intergenic, no info in F-SNP | No leads in HugeNavigator and PubMed (in Dec 2013) |
|  | | |  | rs3990403 | 22 | | | Downstream, no info in F-SNP | No leads in HugeNavigator and PubMed (in Dec 2013) |
| ***TSPO*** | | | Gene expression study |  |  | | |  | Associated with poor breast cancer survival + prostate cancer progression |
|  | | |  | rs6971 | 29 | | | NS coding, 0.83 | No leads in HugeNavigator and PubMed (in Dec 2013) |
| ***RRM2*** | | | Gene expression study |  |  | | |  |  |
|  | | |  | rs6759180* | 29 | | | Intronic, no info in F-SNP | Associated with breast cancer risk |
|  | | |  | rs6741290* | 42 | | | Intronic, no info in F-SNP | Associated with breast cancer risk |
| ***SLC7A11*** | | | Gene expression studies, |  |  | | |  |  |
|  | | |  | rs6838248* | 43 | | | S coding, 0.37 | No leads in HugeNavigator and PubMed (in Dec 2013) |
| ***NFKB1*** | | | Gene expression studies |  |  | | |  |  |
|  | | |  | rs28362491*  (-94 ins/del ATTG) | 30 | | | 5’ near gene, 0.10 | Associated with ovarian cancer risk + prostate cancer risk |
| ***PTGS2*** | | | Gene expression and activity studies |  |  | | |  |  |
|  | | |  | rs5275* | 38 | | | 3-UTR, 0.18 | Associated with breast cancer risk |
| ***NOS2*** | | | Gene expression and activity studies |  |  | | |  |  |
|  | | |  | rs9282801 | 33 | | | Intronic, 0.21 | Associated with prostate cancer risk |
|  | | |  | rs944722* | 49 | | | Intronic, not in F-SNP | Associated with prostate cancer risk |
| ***MGC12965*** | | |  |  |  | | |  |  |
|  | | | Kleinjans GWAS (personal communication) | rs1280350* | 19 | | | Intergenic, 0.51 | No leads in HugeNavigator and PubMed (in Dec 2013) |

* eventually genotyped

1. Duale, N., T. Bjellaas, J. Alexander, G. Becher, M. Haugen, J.E. Paulsen, H. Frandsen, P.T. Olesen, and G. Brunborg (2009) Biomarkers of human exposure to acrylamide and relation to polymorphisms in metabolizing genes. Toxicol Sci 108(1): p. 90-99.

2. Yang, J., L.X. Qian, H.F. Wu, Z.Q. Xu, Y.G. Sui, X.R. Wang, and W. Zhang (2006) Genetic polymorphisms in the cytochrome P450 1A1 and 2E1 genes, smoking, drinking and prostate cancer susceptibility: a case-control study in a Han nationality population in Southern China. Int J Urol 13(6): p. 773-780.

3. Wu, S.H., S.M. Tsai, M.F. Hou, H.S. Lin, L.A. Hou, H. Ma, J.T. Lin, F.L. Yeh, and L.Y. Tsai (2006) Interaction of genetic polymorphisms in cytochrome P450 2E1 and glutathione S-transferase M1 to breast cancer in Taiwanese woman without smoking and drinking habits. Breast Cancer Res Treat 100(1): p. 93-98.

4. Ferreira, P.M., R. Medeiros, A. Vasconcelos, S. Costa, D. Pinto, A. Morais, J. Oliveira, and C. Lopes (2003) Association between CYP2E1 polymorphisms and susceptibility to prostate cancer. Eur J Cancer Prev 12(3): p. 205-211.

5. Clement, F.C., R. Dip, and H. Naegeli (2007) Expression profile of human cells in culture exposed to glycidamide, a reactive metabolite of the heat-induced food carcinogen acrylamide. Toxicology 240(1-2): p. 111-124.

6. Huang, Y.F., M.L. Chen, S.H. Liou, M.F. Chen, S.N. Uang, and K.Y. Wu (2011) Association of CYP2E1, GST and mEH genetic polymorphisms with urinary acrylamide metabolites in workers exposed to acrylamide. Toxicol Lett.

7. Spurdle, A.B., D.M. Purdie, P.M. Webb, X. Chen, A. Green, and G. Chenevix-Trench (2001) The microsomal epoxide hydrolase Tyr113His polymorphism: association with risk of ovarian cancer. Mol Carcinog 30(1): p. 71-78.

8. Goode, E.L., K.L. White, R.A. Vierkant, C.M. Phelan, J.M. Cunningham, J.M. Schildkraut, A. Berchuck, M.C. Larson, B.L. Fridley, J.E. Olson, P.M. Webb, X. Chen, J. Beesley, G. Chenevix-Trench, T.A. Sellers, C. Ovarian Cancer Association, and G. Australian Ovarian Cancer Study (2011) Xenobiotic-Metabolizing gene polymorphisms and ovarian cancer risk. Mol Carcinog 50(5): p. 397-402.

9. Khedhaier, A., E. Hassen, N. Bouaouina, S. Gabbouj, S.B. Ahmed, and L. Chouchane (2008) Implication of Xenobiotic Metabolizing Enzyme gene (CYP2E1, CYP2C19, CYP2D6, mEH and NAT2) polymorphisms in breast carcinoma. BMC Cancer 8: p. 109.

10. Mittal, R.D. and D.L. Srivastava (2007) Cytochrome P4501A1 and microsomal epoxide hydrolase gene polymorphisms: gene-environment interaction and risk of prostate cancer. DNA Cell Biol 26(11): p. 791-798.

11. Pingarilho, M., N.G. Oliveira, C. Martins, B.C. Gomes, A.S. Fernandes, V. Martins, A. Labilloy, J.P. de Lima, J. Rueff, and J.F. Gaspar (2013) Induction of sister chromatid exchange by acrylamide and glycidamide in human lymphocytes: role of polymorphisms in detoxification and DNA-repair genes in the genotoxicity of glycidamide. Mutat Res 752(1-2): p. 1-7.

12. Yang, H.J., S.H. Lee, Y. Jin, J.H. Choi, D.U. Han, C. Chae, M.H. Lee, and C.H. Han (2005) Toxicological effects of acrylamide on rat testicular gene expression profile. Reprod Toxicol 19(4): p. 527-534.

13. Mei, N., L. Guo, J. Tseng, S.L. Dial, W. Liao, and M.G. Manjanatha (2008) Gene expression changes associated with xenobiotic metabolism pathways in mice exposed to acrylamide. Environ Mol Mutagen 49(9): p. 741-745.

14. Kjuus, H., I.L. Hansteen, D. Ryberg, L.O. Goffeng, S. Ovrebo, and V. Skaug (2005) Chromosome aberrations in tunnel workers exposed to acrylamide and N-methylolacrylamide. Scand J Work Environ Health 31(4): p. 300-306.

15. Sen, A., O. Ozgun, E. Arinc, and S. Arslan (2012) Diverse action of acrylamide on cytochrome P450 and glutathione S-transferase isozyme activities, mRNA levels and protein levels in human hepatocarcinoma cells. Cell Biol Toxicol 28(3): p. 175-186.

16. Qiu, L.X., H. Yuan, K.D. Yu, C. Mao, B. Chen, P. Zhan, K. Xue, J. Zhang, and X.C. Hu (2010) Glutathione S-transferase M1 polymorphism and breast cancer susceptibility: a meta-analysis involving 46,281 subjects. Breast Cancer Res Treat 121(3): p. 703-708.

17. Mo, Z., Y. Gao, Y. Cao, F. Gao, and L. Jian (2009) An updating meta-analysis of the GSTM1, GSTT1, and GSTP1 polymorphisms and prostate cancer: a HuGE review. Prostate 69(6): p. 662-688.

18. Baxter, S.W., E.J. Thomas, and I.G. Campbell (2001) GSTM1 null polymorphism and susceptibility to endometriosis and ovarian cancer. Carcinogenesis 22(1): p. 63-65.

19. Oliveira, C., G.J. Lourenco, R.A. Sagarra, S.F. Derchain, J.G. Segalla, and C.S. Lima (2012) Polymorphisms of glutathione S-transferase Mu 1 (GSTM1), Theta 1 (GSTT1), and Pi 1 (GSTP1) genes and epithelial ovarian cancer risk. Dis Markers 33(3): p. 155-159.

20. Ashton, K.A., A. Proietto, G. Otton, I. Symonds, M. McEvoy, J. Attia, M. Gilbert, U. Hamann, and R.J. Scott (2010) Polymorphisms in genes of the steroid hormone biosynthesis and metabolism pathways and endometrial cancer risk. Cancer Epidemiol 34(3): p. 328-337.

21. Song, J., M. Zhao, X. Liu, Y. Zhu, X. Hu, and F. Chen (2013) Protection of cyanidin-3-glucoside against oxidative stress induced by acrylamide in human MDA-MB-231 cells. Food Chem Toxicol 58: p. 306-310.

22. Sergentanis, T.N. and K.P. Economopoulos (2010) GSTT1 and GSTP1 polymorphisms and breast cancer risk: a meta-analysis. Breast Cancer Res Treat 121(1): p. 195-202.

23. Cai, Q., T. Wu, W. Zhang, X. Guo, Z. Shang, N. Jiang, J. Tian, and Y. Niu (2013) Genetic polymorphisms in glutathione S-transferases P1 (GSTP1) Ile105Val and prostate cancer risk: a systematic review and meta-analysis. Tumour Biol 34(6): p. 3913-3922.

24. Chan, Q.K., U.S. Khoo, H.Y. Ngan, C.Q. Yang, W.C. Xue, K.Y. Chan, P.M. Chiu, P.P. Ip, and A.N. Cheung (2005) Single nucleotide polymorphism of pi-class glutathione s-transferase and susceptibility to endometrial carcinoma. Clin Cancer Res 11(8): p. 2981-2985.

25. Mittal, R.D., D.S. Srivastava, A. Mandhani, A. Kumar, and B. Mittal (2004) Polymorphism of GSTM1 and GSTT1 genes in prostate cancer: a study from North India. Indian J Cancer 41(3): p. 115-119.

26. Cunningham, J.M., S.J. Hebbring, S.K. McDonnell, M.S. Cicek, G.B. Christensen, L. Wang, S.J. Jacobsen, J.R. Cerhan, M.L. Blute, D.J. Schaid, and S.N. Thibodeau (2007) Evaluation of genetic variations in the androgen and estrogen metabolic pathways as risk factors for sporadic and familial prostate cancer. Cancer Epidemiol Biomarkers Prev 16(5): p. 969-978.

27. Chen, X.X., R.P. Zhao, L.X. Qiu, H. Yuan, C. Mao, X.C. Hu, and X.M. Guo (2011) Glutathione S-transferase T1 polymorphism is associated with breast cancer susceptibility. Cytokine 56(2): p. 477-480.

28. Karageorgi, S., J. Prescott, J.Y. Wong, I.M. Lee, J.E. Buring, and I. De Vivo (2011) GSTM1 and GSTT1 copy number variation in population-based studies of endometrial cancer risk. Cancer Epidemiol Biomarkers Prev 20(7): p. 1447-1452.

29. Yang, H.P., J. Gonzalez Bosquet, Q. Li, E.A. Platz, L.A. Brinton, M.E. Sherman, J.V. Lacey, Jr., M.M. Gaudet, L.A. Burdette, J.D. Figueroa, J.G. Ciampa, J. Lissowska, B. Peplonska, S.J. Chanock, and M. Garcia-Closas (2010) Common genetic variation in the sex hormone metabolic pathway and endometrial cancer risk: pathway-based evaluation of candidate genes. Carcinogenesis 31(5): p. 827-833.

30. Yu, C.C., S.P. Huang, Y.C. Lee, C.Y. Huang, C.C. Liu, T.C. Hour, C.N. Huang, B.J. You, T.Y. Chang, C.H. Huang, and B.Y. Bao (2013) Molecular markers in sex hormone pathway genes associated with the efficacy of androgen-deprivation therapy for prostate cancer. PLoS One 8(1): p. e54627.

31. Reding, K.W., C.I. Li, N.S. Weiss, C. Chen, C.S. Carlson, D. Duggan, K.E. Thummel, J.R. Daling, and K.E. Malone (2009) Genetic variation in the progesterone receptor and metabolism pathways and hormone therapy in relation to breast cancer risk. Am J Epidemiol 170(10): p. 1241-1249.

32. Kwon, E.M., S.K. Holt, R. Fu, S. Kolb, G. Williams, J.L. Stanford, and E.A. Ostrander (2012) Androgen metabolism and JAK/STAT pathway genes and prostate cancer risk. Cancer Epidemiol 36(4): p. 347-353.

33. He, X.F., W. Wei, S.X. Li, J. Su, Y. Zhang, X.H. Ye, Y. Liu, and W. Wang (2012) Association between the COMT Val158Met polymorphism and breast cancer risk: a meta-analysis of 30,199 cases and 38,922 controls. Mol Biol Rep 39(6): p. 6811-6823.

34. Teng, Y., C. He, X. Zuo, and X. Li (2013) Catechol-O-methyltransferase and cytochrome P-450 1B1 polymorphisms and endometrial cancer risk: a meta-analysis. Int J Gynecol Cancer 23(3): p. 422-430.

35. Ji, Y., J. Olson, J. Zhang, M. Hildebrandt, L. Wang, J. Ingle, Z. Fredericksen, T. Sellers, W. Miller, J.M. Dixon, H. Brauch, M. Eichelbaum, C. Justenhoven, U. Hamann, Y. Ko, T. Bruning, J. Chang-Claude, S. Wang-Gohrke, D. Schaid, and R. Weinshilboum (2008) Breast cancer risk reduction and membrane-bound catechol O-methyltransferase genetic polymorphisms. Cancer Res 68(14): p. 5997-6005.

36. Moreno-Galvan, M., N.E. Herrera-Gonzalez, V. Robles-Perez, J.C. Velasco-Rodriguez, R. Tapia-Conyer, and E. Sarti (2010) Impact of CYP1A1 and COMT genotypes on breast cancer risk in Mexican women: a pilot study. Int J Biol Markers 25(3): p. 157-163.

37. Shin, A., D. Kang, J.Y. Choi, K.M. Lee, S.K. Park, D.Y. Noh, S.H. Ahn, and K.Y. Yoo (2007) Cytochrome P450 1A1 (CYP1A1) polymorphisms and breast cancer risk in Korean women. Exp Mol Med 39(3): p. 361-366.

38. Hirata, H., Y. Hinoda, N. Okayama, Y. Suehiro, K. Kawamoto, N. Kikuno, J.T. Rabban, L.M. Chen, and R. Dahiya (2008) CYP1A1, SULT1A1, and SULT1E1 polymorphisms are risk factors for endometrial cancer susceptibility. Cancer 112(9): p. 1964-1973.

39. Shaik, A.P., K. Jamil, and P. Das (2009) CYP1A1 polymorphisms and risk of prostate cancer: a meta-analysis. Urol J 6(2): p. 78-86.

40. Sergentanis, T.N., K.P. Economopoulos, S. Choussein, and N.F. Vlahos (2012) Cytochrome P450 1A1 (CYP1A1) gene polymorphisms and ovarian cancer risk: a meta-analysis. Mol Biol Rep 39(11): p. 9921-9930.

41. Huang, M., Q. Chen, J. Xiao, X. Zhao, and C. Liu (2012) CYP1A1 Ile462Val is a risk factor for ovarian cancer development. Cytokine 58(1): p. 73-78.

42. Sergentanis, T.N. and K.P. Economopoulos (2010) Four polymorphisms in cytochrome P450 1A1 (CYP1A1) gene and breast cancer risk: a meta-analysis. Breast Cancer Res Treat 122(2): p. 459-469.

43. Chen, C., Y. Huang, Y. Li, Y. Mao, and Y. Xie (2007) Cytochrome P450 1A1 (CYP1A1) T3801C and A2455G polymorphisms in breast cancer risk: a meta-analysis. J Hum Genet 52(5): p. 423-435.

44. Sangrajrang, S., Y. Sato, H. Sakamoto, S. Ohnami, N.M. Laird, T. Khuhaprema, P. Brennan, P. Boffetta, and T. Yoshida (2009) Genetic polymorphisms of estrogen metabolizing enzyme and breast cancer risk in Thai women. Int J Cancer 125(4): p. 837-843.

45. Gulyaeva, L.F., O.N. Mikhailova, V.O. PustyInyak, I.V.t. Kim, A.V. Gerasimov, S.E. Krasilnikov, M.L. Filipenko, and E.V. Pechkovsky (2008) Comparative analysis of SNP in estrogen-metabolizing enzymes for ovarian, endometrial, and breast cancers in Novosibirsk, Russia. Adv Exp Med Biol 617: p. 359-366.

46. Tian, Z., Y.L. Li, L. Zhao, and C.L. Zhang (2013) Role of CYP1A2 1F polymorphism in cancer risk: evidence from a meta-analysis of 46 case-control studies. Gene 524(2): p. 168-174.

47. Mikhailova, O.N., L.F. Gulyaeva, A.V. Prudnikov, A.V. Gerasimov, and S.E. Krasilnikov (2006) Estrogen-metabolizing gene polymorphisms in the assessment of female hormone-dependent cancer risk. Pharmacogenomics J 6(3): p. 189-193.

48. McGrath, M., S.E. Hankinson, L. Arbeitman, G.A. Colditz, D.J. Hunter, and I. De Vivo (2004) Cytochrome P450 1B1 and catechol-O-methyltransferase polymorphisms and endometrial cancer susceptibility. Carcinogenesis 25(4): p. 559-565.

49. Cicek, M.S., X. Liu, G. Casey, and J.S. Witte (2005) Role of androgen metabolism genes CYP1B1, PSA/KLK3, and CYP11alpha in prostate cancer risk and aggressiveness. Cancer Epidemiol Biomarkers Prev 14(9): p. 2173-2177.

50. Holt, S.K., M.A. Rossing, K.E. Malone, S.M. Schwartz, N.S. Weiss, and C. Chen (2007) Ovarian cancer risk and polymorphisms involved in estrogen catabolism. Cancer Epidemiol Biomarkers Prev 16(3): p. 481-489.

51. Goodman, M.T., K. McDuffie, L.N. Kolonel, K. Terada, T.A. Donlon, L.R. Wilkens, C. Guo, and L. Le Marchand (2001) Case-control study of ovarian cancer and polymorphisms in genes involved in catecholestrogen formation and metabolism. Cancer Epidemiol Biomarkers Prev 10(3): p. 209-216.

52. Holt, S.K., E.M. Kwon, R. Fu, S. Kolb, Z. Feng, E.A. Ostrander, and J.L. Stanford (2013) Association of variants in estrogen-related pathway genes with prostate cancer risk. Prostate 73(1): p. 1-10.

53. Johnson, N., K. Walker, L.J. Gibson, N. Orr, E. Folkerd, B. Haynes, C. Palles, B. Coupland, M. Schoemaker, M. Jones, P. Broderick, E. Sawyer, M. Kerin, I.P. Tomlinson, M. Zvelebil, S. Chilcott-Burns, K. Tomczyk, G. Simpson, J. Williamson, S.G. Hillier, G. Ross, R.S. Houlston, A. Swerdlow, A. Ashworth, M. Dowsett, J. Peto, I. Dos Santos Silva, and O. Fletcher (2012) CYP3A variation, premenopausal estrone levels, and breast cancer risk. J Natl Cancer Inst 104(9): p. 657-669.

54. Rodrigues, I.S., H. Kuasne, R. Losi-Guembarovski, P.E. Fuganti, E.P. Gregorio, M.O. Kishima, K. Ito, M.A. de Freitas Rodrigues, and I.M. de Syllos Colus (2011) Evaluation of the influence of polymorphic variants CYP1A1 2B, CYP1B1 2, CYP3A4 1B, GSTM1 0, and GSTT1 0 in prostate cancer. Urol Oncol 29(6): p. 654-663.

55. Zeigler-Johnson, C., T. Friebel, A.H. Walker, Y. Wang, E. Spangler, S. Panossian, M. Patacsil, R. Aplenc, A.J. Wein, S.B. Malkowicz, and T.R. Rebbeck (2004) CYP3A4, CYP3A5, and CYP3A43 genotypes and haplotypes in the etiology and severity of prostate cancer. Cancer Res 64(22): p. 8461-8467.

56. Plummer, S.J., D.V. Conti, P.L. Paris, A.P. Curran, G. Casey, and J.S. Witte (2003) CYP3A4 and CYP3A5 genotypes, haplotypes, and risk of prostate cancer. Cancer Epidemiol Biomarkers Prev 12(9): p. 928-932.

57. Camacho, L., J.R. Latendresse, L. Muskhelishvili, R. Patton, J.F. Bowyer, M. Thomas, and D.R. Doerge (2012) Effects of acrylamide exposure on serum hormones, gene expression, cell proliferation, and histopathology in male reproductive tissues of Fischer 344 rats. Toxicology letters 211(2): p. 135-143.

58. Terry, K., M. McGrath, I.M. Lee, J. Buring, and I. De Vivo (2010) Genetic variation in CYP11A1 and StAR in relation to endometrial cancer risk. Gynecol Oncol 117(2): p. 255-259.

59. Sun, M., X. Yang, C. Ye, W. Xu, G. Yao, J. Chen, and M. Li (2012) Risk-association of CYP11A1 polymorphisms and breast cancer among Han Chinese women in Southern China. Int J Mol Sci 13(4): p. 4896-4905.

60. Olson, S.H., I. Orlow, S. Bayuga, C. Sima, E.V. Bandera, K. Pulick, S. Faulkner, D. Tommasi, D. Egan, P. Roy, H. Wilcox, A. Asya, I. Modica, H. Asad, R. Soslow, and A.G. Zauber (2008) Variants in hormone biosynthesis genes and risk of endometrial cancer. Cancer Causes Control 19(9): p. 955-963.

61. Antognelli, C., L. Mearini, V.N. Talesa, A. Giannantoni, and E. Mearini (2005) Association of CYP17, GSTP1, and PON1 polymorphisms with the risk of prostate cancer. Prostate 63(3): p. 240-251.

62. Setiawan, V.W., F.R. Schumacher, C.A. Haiman, D.O. Stram, D. Albanes, D. Altshuler, G. Berglund, J. Buring, E.E. Calle, F. Clavel-Chapelon, D.G. Cox, J.M. Gaziano, S.E. Hankinson, R.B. Hayes, B.E. Henderson, J. Hirschhorn, R. Hoover, D.J. Hunter, R. Kaaks, L.N. Kolonel, P. Kraft, J. Ma, L. Le Marchand, J. Linseisen, E. Lund, C. Navarro, K. Overvad, D. Palli, P.H. Peeters, M.C. Pike, E. Riboli, M.J. Stampfer, M.J. Thun, R. Travis, D. Trichopoulos, M. Yeager, R.G. Ziegler, H. Spencer Feigelson, and S.J. Chanock (2007) CYP17 genetic variation and risk of breast and prostate cancer from the National Cancer Institute Breast and Prostate Cancer Cohort Consortium (BPC3). Cancer Epidemiol Biomarkers Prev 16(11): p. 2237-2246.

63. Lundin, E., I. Wirgin, A. Lukanova, Y. Afanasyeva, V. Krogh, T. Axelsson, K. Hemminki, T.V. Clendenen, A.A. Arslan, N. Ohlson, S. Sieri, N. Roy, K.L. Koenig, A. Idahl, F. Berrino, P. Toniolo, G. Hallmans, A. Forsti, P. Muti, P. Lenner, R.E. Shore, and A. Zeleniuch-Jacquotte (2012) Selected polymorphisms in sex hormone-related genes, circulating sex hormones and risk of endometrial cancer. Cancer Epidemiol 36(5): p. 445-452.

64. Setiawan, V.W., J.A. Doherty, X.O. Shu, M.R. Akbari, C. Chen, I. De Vivo, A. Demichele, M. Garcia-Closas, M.T. Goodman, C.A. Haiman, S.E. Hankinson, B.E. Henderson, P.L. Horn-Ross, J.V. Lacey, Jr., L. Le Marchand, D.A. Levine, X. Liang, J. Lissowska, G. Lurie, M. McGrath, S.A. Narod, T.R. Rebbeck, G. Ursin, N.S. Weiss, Y.B. Xiang, H.P. Yang, W. Zheng, and S.H. Olson (2009) Two estrogen-related variants in CYP19A1 and endometrial cancer risk: a pooled analysis in the Epidemiology of Endometrial Cancer Consortium. Cancer Epidemiol Biomarkers Prev 18(1): p. 242-247.

65. Goodman, M.T., G. Lurie, P.J. Thompson, K.E. McDuffie, and M.E. Carney (2008) Association of two common single-nucleotide polymorphisms in the CYP19A1 locus and ovarian cancer risk. Endocr Relat Cancer 15(4): p. 1055-1060.

66. Lee, T., M.G. Manjanatha, A. Aidoo, C.L. Moland, W.S. Branham, J.C. Fuscoe, A.A. Ali, and V.G. Desai (2012) Expression analysis of hepatic mitochondria-related genes in mice exposed to acrylamide and glycidamide. J Toxicol Environ Health A 75(6): p. 324-339.

67. Park, J.Y., J.P. Tanner, T.A. Sellers, Y. Huang, C.K. Stevens, N. Dossett, R.A. Shankar, B. Zachariah, R. Heysek, and J. Pow-Sang (2007) Association between polymorphisms in HSD3B1 and UGT2B17 and prostate cancer risk. Urology 70(2): p. 374-379.

68. Setlur, S.R., C.X. Chen, R.R. Hossain, J.S. Ha, V.E. Van Doren, B. Stenzel, E. Steiner, D. Oldridge, N. Kitabayashi, S. Banerjee, J.Y. Chen, G. Schafer, W. Horninger, C. Lee, M.A. Rubin, H. Klocker, and F. Demichelis (2010) Genetic variation of genes involved in dihydrotestosterone metabolism and the risk of prostate cancer. Cancer Epidemiol Biomarkers Prev 19(1): p. 229-239.

69. Beuten, J., J.A. Gelfond, J.L. Franke, K.S. Weldon, A.C. Crandall, T.L. Johnson-Pais, I.M. Thompson, and R.J. Leach (2009) Single and multigenic analysis of the association between variants in 12 steroid hormone metabolism genes and risk of prostate cancer. Cancer Epidemiol Biomarkers Prev 18(6): p. 1869-1880.

70. Feigelson, H.S., D.G. Cox, H.M. Cann, S. Wacholder, R. Kaaks, B.E. Henderson, D. Albanes, D. Altshuler, G. Berglund, F. Berrino, S. Bingham, J.E. Buring, N.P. Burtt, E.E. Calle, S.J. Chanock, F. Clavel-Chapelon, G. Colditz, W.R. Diver, M.L. Freedman, C.A. Haiman, S.E. Hankinson, R.B. Hayes, J.N. Hirschhorn, D. Hunter, L.N. Kolonel, P. Kraft, L. LeMarchand, J. Linseisen, W. Modi, C. Navarro, P.H. Peeters, M.C. Pike, E. Riboli, V.W. Setiawan, D.O. Stram, G. Thomas, M.J. Thun, A. Tjonneland, and D. Trichopoulos (2006) Haplotype analysis of the HSD17B1 gene and risk of breast cancer: a comprehensive approach to multicenter analyses of prospective cohort studies. Cancer Res 66(4): p. 2468-2475.

71. Audet-Walsh, E., J. Bellemare, L. Lacombe, Y. Fradet, V. Fradet, P. Douville, C. Guillemette, and E. Levesque (2012) The impact of germline genetic variations in hydroxysteroid (17-beta) dehydrogenases on prostate cancer outcomes after prostatectomy. Eur Urol 62(1): p. 88-96.

72. Berndt, S.I., N. Chatterjee, W.Y. Huang, S.J. Chanock, R. Welch, E.D. Crawford, and R.B. Hayes (2007) Variant in sex hormone-binding globulin gene and the risk of prostate cancer. Cancer Epidemiol Biomarkers Prev 16(1): p. 165-168.

73. Garcia-Closas, M., L.A. Brinton, J. Lissowska, D. Richesson, M.E. Sherman, N. Szeszenia-Dabrowska, B. Peplonska, R. Welch, M. Yeager, W. Zatonski, and S.J. Chanock (2007) Ovarian cancer risk and common variation in the sex hormone-binding globulin gene: a population-based case-control study. BMC Cancer 7: p. 60.

74. Hein, R., S. Abbas, P. Seibold, R. Salazar, D. Flesch-Janys, and J. Chang-Claude (2012) Polymorphism Thr160Thr in SRD5A1, involved in the progesterone metabolism, modifies postmenopausal breast cancer risk associated with menopausal hormone therapy. Breast Cancer Res Treat 131(2): p. 653-661.

75. Balistreri, C.R., C. Caruso, G. Carruba, V. Miceli, and G. Candore (2011) Genotyping of sex hormone-related pathways in benign and malignant human prostate tissues: data of a preliminary study. OMICS 15(6): p. 369-374.

76. Li, X., Y. Huang, X. Fu, C. Chen, D. Zhang, L. Yan, Y. Xie, Y. Mao, and Y. Li (2011) Meta-analysis of three polymorphisms in the steroid-5-alpha-reductase, alpha polypeptide 2 gene (SRD5A2) and risk of prostate cancer. Mutagenesis 26(3): p. 371-383.

77. Wang, C., W. Tao, Q. Chen, H. Hu, X.Y. Wen, and R. Han (2010) SRD5A2 V89L polymorphism and prostate cancer risk: a meta-analysis. Prostate 70(2): p. 170-178.

78. Lindstrom, S., S.L. Zheng, F. Wiklund, B.A. Jonsson, H.O. Adami, K.A. Balter, A.J. Brookes, J. Sun, B.L. Chang, W. Liu, G. Li, W.B. Isaacs, J. Adolfsson, H. Gronberg, and J. Xu (2006) Systematic replication study of reported genetic associations in prostate cancer: Strong support for genetic variation in the androgen pathway. Prostate 66(16): p. 1729-1743.

79. Yang, G., Y.T. Gao, Q.Y. Cai, X.O. Shu, J.R. Cheng, and W. Zheng (2005) Modifying effects of sulfotransferase 1A1 gene polymorphism on the association of breast cancer risk with body mass index or endogenous steroid hormones. Breast Cancer Res Treat 94(1): p. 63-70.

80. Rebbeck, T.R., A.B. Troxel, Y. Wang, A.H. Walker, S. Panossian, S. Gallagher, E.G. Shatalova, R. Blanchard, G. Bunin, A. DeMichele, S.C. Rubin, M. Baumgarten, M. Berlin, R. Schinnar, J.A. Berlin, and B.L. Strom (2006) Estrogen sulfation genes, hormone replacement therapy, and endometrial cancer risk. J Natl Cancer Inst 98(18): p. 1311-1320.

81. Hasegawa, K., S. Miwa, K. Isomura, K. Tsutsumiuchi, H. Taniguchi, and J. Miwa (2007) Acrylamide-responsive genes in the nematode Caenorhabditis elegans. Toxicol Sci.

82. Deming, S.L., W. Zheng, W.H. Xu, Q. Cai, Z. Ruan, Y.B. Xiang, and X.O. Shu (2008) UGT1A1 genetic polymorphisms, endogenous estrogen exposure, soy food intake, and endometrial cancer risk. Cancer Epidemiol Biomarkers Prev 17(3): p. 563-570.

83. Koutros, S., S.I. Berndt, R. Sinha, X. Ma, N. Chatterjee, M.C. Alavanja, T. Zheng, W.Y. Huang, R.B. Hayes, and A.J. Cross (2009) Xenobiotic metabolizing gene variants, dietary heterocyclic amine intake, and risk of prostate cancer. Cancer Res 69(5): p. 1877-1884.

84. Park, J., L. Chen, L. Ratnashinge, T.A. Sellers, J.P. Tanner, J.H. Lee, N. Dossett, N. Lang, F.F. Kadlubar, C.B. Ambrosone, B. Zachariah, R.V. Heysek, S. Patterson, and J. Pow-Sang (2006) Deletion polymorphism of UDP-glucuronosyltransferase 2B17 and risk of prostate cancer in African American and Caucasian men. Cancer Epidemiol Biomarkers Prev 15(8): p. 1473-1478.

85. Karypidis, A.H., M. Olsson, S.O. Andersson, A. Rane, and L. Ekstrom (2008) Deletion polymorphism of the UGT2B17 gene is associated with increased risk for prostate cancer and correlated to gene expression in the prostate. Pharmacogenomics J 8(2): p. 147-151.

86. Cai, L., W. Huang, and K.C. Chou (2012) Prostate cancer with variants in CYP17 and UGT2B17 genes: a meta-analysis. Protein Pept Lett 19(1): p. 62-69.

87. Eskandari-Nasab, E., M. Hashemi, H. Rezaei, A. Fazaeli, M.A. Mashhadi, S.S. Moghaddam, F. Arbabi, M. Jahantigh, and M. Taheri (2012) Evaluation of UDP-glucuronosyltransferase 2B17 (UGT2B17) and dihydrofolate reductase (DHFR) genes deletion and the expression level of NGX6 mRNA in breast cancer. Mol Biol Rep 39(12): p. 10531-10539.

88. Fernandez, L.P., R.L. Milne, E. Barroso, M. Cuadros, J.I. Arias, A. Ruibal, J. Benitez, and G. Ribas (2006) Estrogen and progesterone receptor gene polymorphisms and sporadic breast cancer risk: a Spanish case-control study. Int J Cancer 119(2): p. 467-471.

89. Hsiao, W.C., K.C. Young, S.L. Lin, and P.W. Lin (2004) Estrogen receptor-alpha polymorphism in a Taiwanese clinical breast cancer population: a case-control study. Breast Cancer Res 6(3): p. R180-186.

90. Sasaki, M., Y. Tanaka, M. Kaneuchi, N. Sakuragi, and R. Dahiya (2002) Polymorphisms of estrogen receptor alpha gene in endometrial cancer. Biochem Biophys Res Commun 297(3): p. 558-564.

91. Ashton, K.A., A. Proietto, G. Otton, I. Symonds, M. McEvoy, J. Attia, M. Gilbert, U. Hamann, and R.J. Scott (2009) Estrogen receptor polymorphisms and the risk of endometrial cancer. BJOG 116(8): p. 1053-1061.

92. Zhou, X., Y. Gu, D.N. Wang, S. Ni, and J. Yan (2013) Eight functional polymorphisms in the estrogen receptor 1 gene and endometrial cancer risk: a meta-analysis. PLoS One 8(4): p. e60851.

93. Wang, Y., M. Cui, and L. Zheng (2012) Genetic polymorphisms in the estrogen receptor-alpha gene and the risk of endometrial cancer: a meta-analysis. Acta Obstet Gynecol Scand 91(8): p. 911-916.

94. Wedren, S., L. Lovmar, K. Humphreys, C. Magnusson, H. Melhus, A.C. Syvanen, A. Kindmark, U. Landegren, M.L. Fermer, F. Stiger, I. Persson, J.A. Baron, and E. Weiderpass (2008) Estrogen receptor alpha gene polymorphism and endometrial cancer risk--a case-control study. BMC Cancer 8: p. 322.

95. Wang, Y.M., Z.W. Liu, J.B. Guo, X.F. Wang, X.X. Zhao, and X. Zheng (2013) ESR1 Gene Polymorphisms and Prostate Cancer Risk: A HuGE Review and Meta-Analysis. PLoS One 8(6): p. e66999.

96. Zhang, L., L. Gu, B. Qian, X. Hao, W. Zhang, Q. Wei, and K. Chen (2009) Association of genetic polymorphisms of ER-alpha and the estradiol-synthesizing enzyme genes CYP17 and CYP19 with breast cancer risk in Chinese women. Breast Cancer Res Treat 114(2): p. 327-338.

97. Wang, J., R. Higuchi, F. Modugno, J. Li, N. Umblas, J. Lee, L.Y. Lui, E. Ziv, J.A. Tice, S.R. Cummings, and B. Rhees (2007) Estrogen receptor alpha haplotypes and breast cancer risk in older Caucasian women. Breast Cancer Res Treat 106(2): p. 273-280.

98. Ding, X., F.M. Cui, S.T. Xu, J.X. Pu, Y.H. Huang, J.L. Zhang, X.D. Wei, J.Q. Hou, and C.Y. Yan (2012) Variants on ESR1 and their association with prostate cancer risk: a meta-analysis. Asian Pac J Cancer Prev 13(8): p. 3931-3936.

99. Mavaddat, N., A.M. Dunning, B.A. Ponder, D.F. Easton, and P.D. Pharoah (2009) Common genetic variation in candidate genes and susceptibility to subtypes of breast cancer. Cancer Epidemiol Biomarkers Prev 18(1): p. 255-259.

100. Yu, K.D., N.Y. Rao, A.X. Chen, L. Fan, C. Yang, and Z.M. Shao (2011) A systematic review of the relationship between polymorphic sites in the estrogen receptor-beta (ESR2) gene and breast cancer risk. Breast Cancer Res Treat 126(1): p. 37-45.

101. Safarinejad, M.R., S. Safarinejad, N. Shafiei, and S. Safarinejad (2012) Estrogen receptors alpha (rs2234693 and rs9340799), and beta (rs4986938 and rs1256049) genes polymorphism in prostate cancer: evidence for association with risk and histopathological tumor characteristics in Iranian men. Mol Carcinog 51 Suppl 1: p. E104-117.

102. Lurie, G., L.R. Wilkens, P.J. Thompson, K.E. McDuffie, M.E. Carney, K.Y. Terada, and M.T. Goodman (2009) Genetic polymorphisms in the estrogen receptor beta (ESR2) gene and the risk of epithelial ovarian carcinoma. Cancer Causes Control 20(1): p. 47-55.

103. Treeck, O., E. Elemenler, C. Kriener, F. Horn, A. Springwald, A. Hartmann, and O. Ortmann (2009) Polymorphisms in the promoter region of ESR2 gene and breast cancer susceptibility. J Steroid Biochem Mol Biol 114(3-5): p. 207-211.

104. Thellenberg-Karlsson, C., S. Lindstrom, B. Malmer, F. Wiklund, K. Augustsson-Balter, H.O. Adami, P. Stattin, M. Nilsson, K. Dahlman-Wright, J.A. Gustafsson, and H. Gronberg (2006) Estrogen receptor beta polymorphism is associated with prostate cancer risk. Clin Cancer Res 12(6): p. 1936-1941.

105. Modugno, F. (2004) Ovarian cancer and polymorphisms in the androgen and progesterone receptor genes: a HuGE review. Am J Epidemiol 159(4): p. 319-335.

106. Pooley, K.A., C.S. Healey, P.L. Smith, P.D. Pharoah, D. Thompson, L. Tee, J. West, C. Jordan, D.F. Easton, B.A. Ponder, and A.M. Dunning (2006) Association of the progesterone receptor gene with breast cancer risk: a single-nucleotide polymorphism tagging approach. Cancer Epidemiol Biomarkers Prev 15(4): p. 675-682.

107. Xu, W.H., J.R. Long, W. Zheng, Z.X. Ruan, Q. Cai, J.R. Cheng, Y.B. Xiang, and X.O. Shu (2009) Association of the progesterone receptor gene with endometrial cancer risk in a Chinese population. Cancer 115(12): p. 2693-2700.

108. Pearce, C.L., A.H. Wu, S.A. Gayther, A.E. Bale, S. Australian Cancer, G. Australian Cancer Study, P.A. Beck, J. Beesley, S. Chanock, D.W. Cramer, R. DiCioccio, R. Edwards, Z.S. Fredericksen, M. Garcia-Closas, E.L. Goode, A.C. Green, L.C. Hartmann, E. Hogdall, S.K. Kjaer, J. Lissowska, V. McGuire, F. Modugno, K. Moysich, R.B. Ness, S.J. Ramus, H.A. Risch, T.A. Sellers, H. Song, D.O. Stram, K.L. Terry, P.M. Webb, D.C. Whiteman, A.S. Whittemore, W. Zheng, P.D. Pharoah, G. Chenevix-Trench, M.C. Pike, J. Schildkraut, A. Berchuck, and C. Ovarian Cancer Association (2008) Progesterone receptor variation and risk of ovarian cancer is limited to the invasive endometrioid subtype: results from the Ovarian Cancer Association Consortium pooled analysis. Br J Cancer 98(2): p. 282-288.

109. Leite, D.B., M.G. Junqueira, C.V. de Carvalho, A.M. Massad-Costa, W.J. Goncalves, S.M. Nicolau, L.A. Lopes, E.C. Baracat, and I.D. da Silva (2008) Progesterone receptor (PROGINS) polymorphism and the risk of ovarian cancer. Steroids 73(6): p. 676-680.

110. Rockwell, L.C., E.J. Rowe, K. Arnson, F. Jackson, A. Froment, P. Ndumbe, B. Seck, R. Jackson, and J.G. Lorenz (2012) Worldwide distribution of allelic variation at the progesterone receptor locus and the incidence of female reproductive cancers. Am J Hum Biol 24(1): p. 42-51.

111. Johnatty, S.E., A.B. Spurdle, J. Beesley, X. Chen, J.L. Hopper, D.L. Duffy, G. Chenevix-Trench, and C. Kathleen Cuningham Consortium for Research in Familial Breast (2008) Progesterone receptor polymorphisms and risk of breast cancer: results from two Australian breast cancer studies. Breast Cancer Res Treat 109(1): p. 91-99.

112. Junqueira, M.G., I.D. da Silva, N.C. Nogueira-de-Souza, C.V. Carvalho, D.B. Leite, M.T. Gomes, E.C. Baracat, L.A. Lopes, S.M. Nicolau, and W.J. Goncalves (2007) Progesterone receptor (PROGINS) polymorphism and the risk of endometrial cancer development. Int J Gynecol Cancer 17(1): p. 229-232.

113. Hayes, V.M., G. Severi, S.A. Eggleton, E.J. Padilla, M.C. Southey, R.L. Sutherland, J.L. Hopper, and G.G. Giles (2005) The E211 G>A androgen receptor polymorphism is associated with a decreased risk of metastatic prostate cancer and androgenetic alopecia. Cancer Epidemiol Biomarkers Prev 14(4): p. 993-996.

114. Yang, H.P., M. Garcia-Closas, J.V. Lacey, Jr., L.A. Brinton, J. Lissowska, B. Peplonska, S. Chanock, and M.M. Gaudet (2009) Genetic variation in the androgen receptor gene and endometrial cancer risk. Cancer Epidemiol Biomarkers Prev 18(2): p. 585-589.

115. McCullough, L.E., R.M. Santella, R.J. Cleveland, P.T. Bradshaw, R.C. Millikan, K.E. North, A.F. Olshan, S.M. Eng, C.B. Ambrosone, J. Ahn, S.E. Steck, S.L. Teitelbaum, A.I. Neugut, and M.D. Gammon (2012) Polymorphisms in oxidative stress genes, physical activity, and breast cancer risk. Cancer Causes Control 23(12): p. 1949-1958.

116. Karunasinghe, N., D.Y. Han, M. Goudie, S. Zhu, K. Bishop, A. Wang, H. Duan, K. Lange, S. Ko, R. Medhora, S.T. Kan, J. Masters, and L.R. Ferguson (2012) Prostate disease risk factors among a New Zealand cohort. J Nutrigenet Nutrigenomics 5(6): p. 339-351.

117. Tefik, T., C. Kucukgergin, O. Sanli, T. Oktar, S. Seckin, and C. Ozsoy (2013) Manganese superoxide dismutase Ile58Thr, catalase C-262T and myeloperoxidase G-463A gene polymorphisms in patients with prostate cancer: relation to advanced and metastatic disease. BJU Int 112(4): p. E406-414.

118. Li, Y., C.B. Ambrosone, M.J. McCullough, J. Ahn, V.L. Stevens, M.J. Thun, and C.C. Hong (2009) Oxidative stress-related genotypes, fruit and vegetable consumption and breast cancer risk. Carcinogenesis 30(5): p. 777-784.

119. Quick, S.K., P.G. Shields, J. Nie, M.E. Platek, S.E. McCann, A.D. Hutson, M. Trevisan, D. Vito, R. Modali, T.A. Lehman, M. Seddon, S.B. Edge, C. Marian, P. Muti, and J.L. Freudenheim (2008) Effect modification by catalase genotype suggests a role for oxidative stress in the association of hormone replacement therapy with postmenopausal breast cancer risk. Cancer Epidemiol Biomarkers Prev 17(5): p. 1082-1087.

120. Cebrian, A., P.D. Pharoah, S. Ahmed, P.L. Smith, C. Luccarini, R. Luben, K. Redman, H. Munday, D.F. Easton, A.M. Dunning, and B.A. Ponder (2006) Tagging single-nucleotide polymorphisms in antioxidant defense enzymes and susceptibility to breast cancer. Cancer Res 66(2): p. 1225-1233.

121. Ding, G., F. Liu, B. Shen, C. Feng, J. Xu, and Q. Ding (2012) The association between polymorphisms in prooxidant or antioxidant enzymes (myeloperoxidase, SOD2, and CAT) and genes and prostate cancer risk in the Chinese population of Han nationality. Clin Genitourin Cancer 10(4): p. 251-255.

122. Ravn-Haren, G., A. Olsen, A. Tjonneland, L.O. Dragsted, B.A. Nexo, H. Wallin, K. Overvad, O. Raaschou-Nielsen, and U. Vogel (2006) Associations between GPX1 Pro198Leu polymorphism, erythrocyte GPX activity, alcohol consumption and breast cancer risk in a prospective cohort study. Carcinogenesis 27(4): p. 820-825.

123. Tsai, S.M., S.H. Wu, M.F. Hou, Y.L. Chen, H. Ma, and L.Y. Tsai (2012) Oxidative stress-related enzyme gene polymorphisms and susceptibility to breast cancer in non-smoking, non-alcohol-consuming Taiwanese women: a case-control study. Ann Clin Biochem 49(Pt 2): p. 152-158.

124. Arsova-Sarafinovska, Z., N. Matevska, A. Eken, D. Petrovski, S. Banev, S. Dzikova, V. Georgiev, A. Sikole, O. Erdem, A. Sayal, A. Aydin, and A.J. Dimovski (2009) Glutathione peroxidase 1 (GPX1) genetic polymorphism, erythrocyte GPX activity, and prostate cancer risk. Int Urol Nephrol 41(1): p. 63-70.

125. Steinbrecher, A., C. Meplan, J. Hesketh, L. Schomburg, T. Endermann, E. Jansen, B. Akesson, S. Rohrmann, and J. Linseisen (2010) Effects of selenium status and polymorphisms in selenoprotein genes on prostate cancer risk in a prospective study of European men. Cancer Epidemiol Biomarkers Prev 19(11): p. 2958-2968.

126. Geybels, M.S., P.A. van den Brandt, L.J. Schouten, F.J. van Schooten, S.G. van Breda, M.P. Rayman, F.R. Green, and B.A. Verhage (2014) Selenoprotein gene variants, toenail selenium levels, and risk for advanced prostate cancer. J Natl Cancer Inst 106(3): p. dju003.

127. Sadek, I.A. (1989) Short-term studies of the effect of acrylamide on the testes of the Egyptian toad. Folia Morphol (Praha) 37(4): p. 427-430.

128. Abe, M., W. Xie, M.M. Regan, I.B. King, M.J. Stampfer, P.W. Kantoff, W.K. Oh, and J.M. Chan (2011) Single-nucleotide polymorphisms within the antioxidant defence system and associations with aggressive prostate cancer. BJU Int 107(1): p. 126-134.

129. Tamimi, R.M., S.E. Hankinson, D. Spiegelman, G.A. Colditz, and D.J. Hunter (2004) Manganese superoxide dismutase polymorphism, plasma antioxidants, cigarette smoking, and risk of breast cancer. Cancer Epidemiol Biomarkers Prev 13(6): p. 989-996.

130. Cai, Q., X.O. Shu, W. Wen, J.R. Cheng, Q. Dai, Y.T. Gao, and W. Zheng (2004) Genetic polymorphism in the manganese superoxide dismutase gene, antioxidant intake, and breast cancer risk: results from the Shanghai Breast Cancer Study. Breast Cancer Res 6(6): p. R647-655.

131. Bica, C.G., L.L. de Moura da Silva, N.V. Toscani, I.B. da Cruz, G. Sa, M.S. Graudenz, and C.G. Zettler (2009) MnSOD gene polymorphism association with steroid-dependent cancer. Pathol Oncol Res 15(1): p. 19-24.

132. Kang, D., K.M. Lee, S.K. Park, S.I. Berndt, U. Peters, D. Reding, N. Chatterjee, R. Welch, S. Chanock, W.Y. Huang, and R.B. Hayes (2007) Functional variant of manganese superoxide dismutase (SOD2 V16A) polymorphism is associated with prostate cancer risk in the prostate, lung, colorectal, and ovarian cancer study. Cancer Epidemiol Biomarkers Prev 16(8): p. 1581-1586.

133. Mao, C., L.X. Qiu, P. Zhan, K. Xue, H. Ding, F.B. Du, J. Li, and Q. Chen (2010) MnSOD Val16Ala polymorphism and prostate cancer susceptibility: a meta-analysis involving 8,962 subjects. J Cancer Res Clin Oncol 136(7): p. 975-979.

134. Olson, S.H., M.D. Carlson, H. Ostrer, S. Harlap, A. Stone, M. Winters, and C.B. Ambrosone (2004) Genetic variants in SOD2, MPO, and NQO1, and risk of ovarian cancer. Gynecol Oncol 93(3): p. 615-620.

135. Seibold, P., R. Hein, P. Schmezer, P. Hall, J. Liu, N. Dahmen, D. Flesch-Janys, O. Popanda, and J. Chang-Claude (2011) Polymorphisms in oxidative stress-related genes and postmenopausal breast cancer risk. Int J Cancer 129(6): p. 1467-1476.

136. Yuan, W., L. Xu, W. Chen, L. Wang, Z. Fu, D. Pang, and D. Li (2011) Evidence on the association between NQO1 Pro187Ser polymorphism and breast cancer risk in the current studies: a meta-analysis. Breast Cancer Res Treat 125(2): p. 467-472.

137. Mandal, R.K., K. Nissar, and R.D. Mittal (2012) Genetic variants in metabolizing genes NQO1, NQO2, MTHFR and risk of prostate cancer: a study from North India. Mol Biol Rep 39(12): p. 11145-11152.

138. Fowke, J.H., X.O. Shu, Q. Dai, F. Jin, Q. Cai, Y.T. Gao, and W. Zheng (2004) Oral contraceptive use and breast cancer risk: modification by NAD(P)H:quinone oxoreductase (NQO1) genetic polymorphisms. Cancer Epidemiol Biomarkers Prev 13(8): p. 1308-1315.

139. Lin, C.Y., H.L. Lee, Y.C. Chen, G.W. Lien, L.Y. Lin, L.L. Wen, C.C. Liao, K.L. Chien, F.C. Sung, P.C. Chen, and T.C. Su (2013) Positive association between urinary levels of 8-hydroxydeoxyguanosine and the acrylamide metabolite N-acetyl-S-(propionamide)-cysteine in adolescents and young adults. J Hazard Mater 261: p. 372-377.

140. Yuan, W., L. Xu, Y. Feng, Y. Yang, W. Chen, J. Wang, D. Pang, and D. Li (2010) The hOGG1 Ser326Cys polymorphism and breast cancer risk: a meta-analysis. Breast Cancer Res Treat 122(3): p. 835-842.

141. Cincin, Z.B., A.C. Iyibozkurt, S.B. Kuran, and B. Cakmakoglu (2012) DNA repair gene variants in endometrial carcinoma. Med Oncol 29(4): p. 2949-2954.

142. Zhu, S., H. Zhang, Y. Tang, and J. Wang (2012) Polymorphisms in XPD and hOGG1 and prostate cancer risk: a meta-analysis. Urol Int 89(2): p. 233-240.

143. Pingarilho, M., N.G. Oliveira, C. Martins, A.S. Fernandes, J.P. de Lima, J. Rueff, and J.F. Gaspar (2012) Genetic polymorphisms in detoxification and DNA repair genes and susceptibility to glycidamide-induced DNA damage. J Toxicol Environ Health A 75(13-15): p. 920-933.

144. Wu, K., D. Su, K. Lin, J. Luo, and W.W. Au (2011) XRCC1 Arg399Gln gene polymorphism and breast cancer risk: a meta-analysis based on case-control studies. Asian Pac J Cancer Prev 12(9): p. 2237-2243.

145. Huang, Y., L. Li, and L. Yu (2009) XRCC1 Arg399Gln, Arg194Trp and Arg280His polymorphisms in breast cancer risk: a meta-analysis. Mutagenesis 24(4): p. 331-339.

146. Chen, L., C.B. Ambrosone, J. Lee, T.A. Sellers, J. Pow-Sang, and J.Y. Park (2006) Association between polymorphisms in the DNA repair genes XRCC1 and APE1, and the risk of prostate cancer in white and black Americans. J Urol 175(1): p. 108-112; discussion 112.

147. Samulak, D., H. Romanowicz-Makowska, B. Smolarz, A. Kulig, and S. Sporny (2011) Association between Arg399Gln polymorphism of X-ray repair cross-complementing 1 (XRCC1) gene and sporadic endometrial cancer in the Polish population. Eur J Gynaecol Oncol 32(5): p. 491-495.

148. He, J., T.Y. Shi, M.L. Zhu, M.Y. Wang, Q.X. Li, and Q.Y. Wei (2013) Associations of Lys939Gln and Ala499Val polymorphisms of the XPC gene with cancer susceptibility: a meta-analysis. Int J Cancer 133(8): p. 1765-1775.

149. Weiss, J.M., N.S. Weiss, C.M. Ulrich, J.A. Doherty, L.F. Voigt, and C. Chen (2005) Interindividual variation in nucleotide excision repair genes and risk of endometrial cancer. Cancer Epidemiol Biomarkers Prev 14(11 Pt 1): p. 2524-2530.

150. Galiegue, S., P. Casellas, A. Kramar, N. Tinel, and J. Simony-Lafontaine (2004) Immunohistochemical assessment of the peripheral benzodiazepine receptor in breast cancer and its relationship with survival. Clin Cancer Res 10(6): p. 2058-2064.

151. Fafalios, A., A. Akhavan, A.V. Parwani, R.R. Bies, K.J. McHugh, and B.R. Pflug (2009) Translocator protein blockade reduces prostate tumor growth. Clin Cancer Res 15(19): p. 6177-6184.

152. Olson, J.E., X. Wang, E.L. Goode, V.S. Pankratz, Z.S. Fredericksen, R.A. Vierkant, P.D. Pharoah, J.R. Cerhan, and F.J. Couch (2010) Variation in genes required for normal mitosis and risk of breast cancer. Breast Cancer Res Treat 119(2): p. 423-430.

153. Ehlers, A., D. Lenze, H. Broll, J. Zagon, M. Hummel, and A. Lampen (2013) Dose dependent molecular effects of acrylamide and glycidamide in human cancer cell lines and human primary hepatocytes. Toxicol Lett 217(2): p. 111-120.

154. Hochstenbach, K., D.M. van Leeuwen, H. Gmuender, R.W. Gottschalk, M. Lovik, B. Granum, U. Nygaard, E. Namork, M. Kirsch-Volders, I. Decordier, K. Vande Loock, H. Besselink, M. Tornqvist, H. von Stedingk, P. Rydberg, J.C. Kleinjans, H. van Loveren, and J.H. van Delft (2012) Global gene expression analysis in cord blood reveals gender-specific differences in response to carcinogenic exposure in utero. Cancer epidemiology, biomarkers & prevention : a publication of the American Association for Cancer Research, cosponsored by the American Society of Preventive Oncology 21(10): p. 1756-1767.

155. Shan, X., Y. Li, X. Meng, P. Wang, P. Jiang, and Q. Feng (2014) Curcumin and (-)-epigallocatechin-3-gallate attenuate acrylamide-induced proliferation in HepG2 cells. Food Chem Toxicol 66: p. 194-202.

156. Fan, Y., W. Yu, P. Ye, H. Wang, Z. Wang, Q. Meng, Y. Duan, X. Liang, and W. An (2011) NFKB1 insertion/deletion promoter polymorphism increases the risk of advanced ovarian cancer in a Chinese population. DNA Cell Biol 30(4): p. 241-245.

157. Huo, Z.H., H.J. Zhong, Y.S. Zhu, B. Xing, and H. Tang (2013) Roles of functional NFKB1 and beta-TrCP insertion/deletion polymorphisms in mRNA expression and epithelial ovarian cancer susceptibility. Genet Mol Res 12(3): p. 3435-3443.

158. Zhang, P., Q. Wei, X. Li, K. Wang, H. Zeng, H. Bu, and H. Li (2009) A functional insertion/deletion polymorphism in the promoter region of the NFKB1 gene increases susceptibility for prostate cancer. Cancer Genet Cytogenet 191(2): p. 73-77.

159. Kopp, T.I., S. Friis, J. Christensen, A. Tjonneland, and U. Vogel (2013) Polymorphisms in genes related to inflammation, NSAID use, and the risk of prostate cancer among Danish men. Cancer Genet 206(7-8): p. 266-278.

160. Lyn-Cook, L.E., Jr., E. Tareke, B. Word, A. Starlard-Davenport, B.D. Lyn-Cook, and G.J. Hammons (2011) Food contaminant acrylamide increases expression of Cox-2 and nitric oxide synthase in breast epithelial cells. Toxicol Ind Health 27(1): p. 11-18.

161. Lim, T.G., B.K. Lee, J.Y. Kwon, S.K. Jung, and K.W. Lee (2011) Acrylamide up-regulates cyclooxygenase-2 expression through the MEK/ERK signaling pathway in mouse epidermal cells. Food Chem Toxicol 49(6): p. 1249-1254.

162. Langsenlehner, U., B. Yazdani-Biuki, T. Eder, W. Renner, T.C. Wascher, B. Paulweber, W. Weitzer, H. Samonigg, and P. Krippl (2006) The cyclooxygenase-2 (PTGS2) 8473T>C polymorphism is associated with breast cancer risk. Clin Cancer Res 12(4): p. 1392-1394.

163. Fawzy, M.S., N.M. Aly, S.M. Shalaby, W.H. El-Sawy, and R.S. Abdul-Maksoud (2013) Cyclooxygenase-2 169C>G and 8473T>C gene polymorphisms and prostaglandin E2 level in breast cancer: a case-control study. Gene 527(2): p. 601-605.

164. Zhu, W., B.B. Wei, X. Shan, and P. Liu (2010) -765G>C and 8473T>C polymorphisms of COX-2 and cancer risk: a meta-analysis based on 33 case-control studies. Mol Biol Rep 37(1): p. 277-288.

165. Kim, K. (2005) Effect of subchronic acrylamide exposure on the expression of neuronal and inducible nitric oxide synthase in rat brain. J Biochem Mol Toxicol 19(3): p. 162-168.

166. Lee, K.M., D. Kang, S.K. Park, S.I. Berndt, D. Reding, N. Chatterjee, S. Chanock, W.Y. Huang, and R.B. Hayes (2009) Nitric oxide synthase gene polymorphisms and prostate cancer risk. Carcinogenesis 30(4): p. 621-625.

Supplemental Table 2: Description of SNPs and their genotype frequencies in the subcohort (participants with sample call rate ≥95% only)

|  | | | | | | | **Genotypes** | | | | | |  |
| --- | --- | --- | --- | --- | --- | --- | --- | --- | --- | --- | --- | --- | --- |
|  |  |  |  |  |  |  | **n** | | | **%** | | | **HWE†** |
| **SNP ID** | **Gene** | **Chr. Location** | **Ref. alleles wild type/variant** | **Minor Allele NLCS** | **MAF NLCS** | **SNPcall rate** | **11*** | **12*** | **22*** | **11** | **12** | **22** | **P value** |
| **Acrylamide metabolism** |  |  |  |  |  |  |  |  |  |  |  |  |  |
| rs6413432 | *CYP2E1* | 10q26.3 | T/A | A | 9.0 | 99.9 | 1436 | 295 | 9 | 82.5 | 17.0 | 0.5 | 0.14 |
| rs915906 | *CYP2E1* | 10q26.3 | T/C | C | 14.9 | 99.9 | 1255 | 451 | 33 | 72.2 | 25.9 | 1.9 | 0.30 |
| rs2480258 | *CYP2E1* | 10q26.3 | G/A | A | 19.0 | 100 | 1129 | 563 | 49 | 64.8 | 32.3 | 2.8 | 0.03 |
| rs1051740 | *EPHX1* | 1q42.1 | T/C | C | 31.1 | 100 | 833 | 734 | 174 | 47.8 | 42.2 | 10.0 | 0.51 |
| rs4715354 | *GSTA5* | 6p12.2 | A/G | G | 48.4 | 100 | 458 | 879 | 404 | 26.3 | 50.5 | 23.2 | 0.65 |
| rs1695 | *GSTP1* | 11q13 | A/G | G | 35.9 | 100 | 706 | 821 | 214 | 40.6 | 47.2 | 12.3 | 0.30 |
| **Sex steroid metabolism** |  |  |  |  |  |  |  |  |  |  |  |  |  |
| rs11252859 | *AKR1C1* | 10p15-p14 | C/T | T | 36.3 | 99.5 | 716 | 773 | 243 | 41.3 | 44.6 | 14.0 | 0.14 |
| rs7741 | *AKR1C2* | 10p15-p14 | G/A | G | 29.3 | 36.7 | 19 | 464 | 156 | 3.0 | 72.6 | 24.4 | <0.001 |
| rs11252887 | *AKR1C2* | 10p15-p14 | C/T | T | 28.3 | 99.3 | 874 | 695 | 135 | 51.3 | 40.8 | 7.9 | 0.85 |
| rs737865 | *COMT* | 22q11.21 | T/C | C | 25.8 | 100 | 953 | 678 | 110 | 54.7 | 38.9 | 6.3 | 0.47 |
| rs4646903 | *CYP1A1* | 15q24.1 | T/C | C | 8.3 | 98.3 | 1429 | 283 | 0 | 83.5 | 16.5 | 0.0 | <0.001 |
| rs2472299 | *CYP1A2* | 15q24.1 | G/A | A | 28.2 | 99.9 | 905 | 688 | 146 | 52.0 | 39.6 | 8.4 | 0.35 |
| rs1056827 | *CYP1B1* | 2p22.2 | G/T | T | 27.1 | 99.8 | 926 | 682 | 130 | 53.3 | 39.2 | 7.5 | 0.77 |
| rs1056836 | *CYP1B1* | 2p22.2 | C/G | G | 44.6 | 99.9 | 546 | 836 | 358 | 31.4 | 48.0 | 20.6 | 0.25 |
| rs3825944 | *CYP11A1* | 15q23-q24 | C/T | T | 16.5 | 100 | 1219 | 468 | 54 | 70.0 | 26.9 | 3.1 | 0.27 |
| rs2959008 | *CYP11A1* | 15q23-q24 | C/T | T | 33.8 | 99.9 | 764 | 774 | 201 | 43.9 | 44.5 | 11.6 | 0.82 |
| rs7173655 | *CYP11A1* | 15q23-q24 | T/C | C | 32.1 | 100 | 813 | 739 | 189 | 46.7 | 42.4 | 10.9 | 0.28 |
| rs4919682 | *CYP17A1* | 10q24.3 | C/T | T | 29.5 | 100 | 851 | 753 | 137 | 48.9 | 43.3 | 7.9 | 0.10 |
| rs4919687 | *CYP17A1* | 10q24.3 | G/A | A | 30.5 | 99.9 | 827 | 763 | 150 | 47.5 | 43.9 | 8.6 | 0.16 |
| rs743572 | *CYP17A1* | 10q24.3 | A/G | G | 39.9 | 99.9 | 625 | 843 | 272 | 35.9 | 48.4 | 15.6 | 0.66 |
| rs11632903 | *CYP19A1* | 15q21.1 | C/T | T | 44.3 | 100 | 546 | 846 | 349 | 31.4 | 48.6 | 20.0 | 0.52 |
| rs4659175 | *HSD3B1/B2* | 1p13.1 | C/T | T | 32.3 | 99.9 | 798 | 759 | 183 | 45.9 | 43.6 | 10.5 | 0.90 |
| rs1047303 | *HSD3B1/B2* | 1p13.1 | A/C | C | 31.6 | 99.9 | 800 | 782 | 158 | 46.0 | 44.9 | 9.1 | 0.09 |
| rs10923823 | *HSD3B1/B2* | 1p13.1 | C/T | C | 45.0 | 100 | 540 | 836 | 365 | 31.0 | 48.0 | 21.0 | 0.21 |
| rs6203 | *HSD3B1/B2* | 1p13.1 | C/T | T | 42.0 | 100 | 568 | 853 | 295 | 33.1 | 49.7 | 17.2 | 0.41 |
| rs6428830 | *HSD3B1/B2* | 1p13.1 | G/A | A | 29.8 | 99.9 | 833 | 743 | 139 | 48.6 | 43.3 | 8.1 | 0.13 |
| rs7546652 | *HSD3B1/B2* | 1p13.1 | T/C | C | 45.0 | 99.9 | 540 | 835 | 365 | 31.0 | 48.0 | 21.0 | 0.20 |
| rs2253502 | *HSD17B3* | 9q22 | T/C | C | 21.3 | 71.9 | 776 | 416 | 59 | 62.0 | 33.3 | 4.7 | 0.73 |
| rs2257157 | *HSD17B3* | 9q22 | T/C | C | 47.8 | 99.8 | 459 | 896 | 383 | 26.4 | 51.6 | 22.0 | 0.17 |
| rs6259 | *SHBG* | 17p13.1 | G/A | A | 11.2 | 99.4 | 1362 | 349 | 19 | 78.7 | 20.2 | 1.1 | 0.52 |
| rs8192120 | *SRD5A1* | 5p15 | C/A | A | 37.2 | 99.9 | 687 | 811 | 242 | 39.5 | 46.6 | 13.9 | 0.91 |
| rs824811 | *SRD5A1* | 5p15 | T/C | C | 23.2 | 100 | 1029 | 617 | 95 | 59.1 | 35.4 | 5.5 | 0.84 |
| rs1042157 | *SULT1A1* | 16p12.1 | C/T | T | 38.8 | 99.7 | 642 | 839 | 254 | 37.0 | 48.4 | 14.6 | 0.45 |
| rs6839 | *SULT1A1* | 16p12.1 | A/G | G | 34.6 | 99.3 | 765 | 730 | 234 | 44.2 | 42.2 | 13.5 | 0.005 |
| rs3736599 | *SULT1E1* | 4q13.1 | G/A | A | 9.5 | 99.9 | 1425 | 298 | 16 | 81.9 | 17.1 | 0.9 | 0.92 |
| rs2070959 | *UGT1A6-10* | 2q37 | A/G | G | 32.3 | 99.9 | 788 | 780 | 172 | 45.3 | 44.8 | 9.9 | 0.30 |
| **Nuclear receptors** |  |  |  |  |  |  |  |  |  |  |  |  |  |
| rs2234693 | *ESR1* | 6q25.1 | T/C | C | 48.9 | 77.6 | 361 | 659 | 331 | 26.7 | 48.8 | 24.5 | 0.38 |
| rs2987983 | *ESR2* | 14q23.2 | T/C | C | 32.5 | 99.9 | 817 | 716 | 207 | 47.0 | 41.1 | 11.9 | 0.01 |
| rs4986938 | *ESR2* | 14q23.2 | G/A | A | 36.1 | 99.9 | 720 | 784 | 235 | 41.4 | 45.1 | 13.5 | 0.35 |
| rs660149 | *PGR* | 11q22-q23 | C/G | G | 26.0 | 100 | 950 | 675 | 116 | 54.6 | 38.8 | 6.7 | 0.79 |
| **Oxidative stress pathway** | | | | | | | | | | | | | |
| rs1001179 | *CAT* | 11p13 | G/A | A | 21.0 | 97.5 | 1074 | 535 | 89 | 63.3 | 31.5 | 5.2 | 0.04 |
| rs511895 | *CAT* | 11p13 | A/G | A | 41.3 | 100 | 584 | 875 | 282 | 33.5 | 50.3 | 16.2 | 0.13 |
| rs3448 | *GPX1* | 3p21.3 | C/T | T | 27.0 | 99.9 | 923 | 696 | 121 | 53.0 | 40.0 | 7.0 | 0.51 |
| rs1800566 | *NQO1* | 16q22.1 | C/T | T | 18.7 | 99.9 | 1146 | 538 | 56 | 65.9 | 30.9 | 3.2 | 0.46 |
| rs10432782 | *SOD1* | 21q22.11 | T/G | G | 11.4 | 100 | 1367 | 350 | 24 | 78.5 | 20.1 | 1.4 | 0.77 |
| rs4880 | *SOD2* | 6q25.3 | T/C | C | 49.1 | 100 | 445 | 882 | 414 | 25.6 | 50.7 | 23.8 | 0.57 |
| rs5746136 | *SOD2* | 6q25.3 | G/A | A | 29.6 | 100 | 872 | 708 | 161 | 50.1 | 40.7 | 9.2 | 0.32 |
| rs2301241 | *TXN* | 9q31 | C/T | C | 38.9 | 99.9 | 640 | 845 | 255 | 36.8 | 48.6 | 14.7 | 0.38 |
| **DNA repair** |  |  |  |  |  |  |  |  |  |  |  |  |  |
| rs1052133 | *OGG1* | 3p26.2 | C/G | G | 22.2 | 100 | 1055 | 600 | 86 | 60.6 | 34.5 | 4.9 | 0.95 |
| rs3219489 | *MUTYH* | 1p34.1 | G/C | C | 24.1 | 99.8 | 1005 | 628 | 104 | 57.9 | 36.2 | 6.0 | 0.65 |
| rs2228000 | *XPC* | 3p25 | C/T | T | 26.4 | 100 | 955 | 653 | 133 | 54.9 | 37.5 | 7.6 | 0.15 |
| rs2228001 | *XPC* | 3p25 | A/C | C | 39.3 | 100 | 633 | 846 | 262 | 36.4 | 48.6 | 15.0 | 0.45 |
| rs25487 | *XRCC1* | 19q13.2 | A/G | A | 35.6 | 99.9 | 721 | 799 | 220 | 41.4 | 45.9 | 12.6 | 0.95 |
| **Gene expression acrylamide/GWAS acrylamide/other** | | | | | | | | | | | | | |
| rs1280350 | *MGC12965* | 11q13.4 | G/T | T | 17.2 | 100 | 1195 | 494 | 52 | 68.6 | 28.4 | 3.0 | 0.91 |
| rs28362491 | *NFKB1* | 4q24 | ATTG/DEL | DEL | 40.2 | 99.9 | 633 | 815 | 291 | 36.4 | 46.9 | 16.7 | 0.30 |
| rs944722 | *NOS2* | 17q11.2-q12 | T/C | C | 39.1 | 96.7 | 646 | 757 | 280 | 38.4 | 45.0 | 16.6 | 0.02 |
| rs5275 | *PTGS2* | 1q25.2-q25.3 | T/C | C | 31.7 | 99.9 | 807 | 763 | 170 | 46.4 | 43.9 | 9.8 | 0.60 |
| rs6741290 | *RRM2* | 2p25-p24 | C/T | T | 42.7 | 99.9 | 576 | 842 | 321 | 33.1 | 48.4 | 18.5 | 0.67 |
| rs6759180 | *RRM2* | 2p25-p24 | A/G | G | 26.4 | 99.1 | 937 | 665 | 124 | 54.3 | 38.5 | 7.2 | 0.69 |
| rs6838248 | *SLC7A11* | 4q28-q32 | C/G | C | 46.6 | 99.9 | 394 | 835 | 511 | 22.6 | 48.0 | 29.4 | 0.13 |

* 11: wildtype/wildtype, 12: wildtype/variant, 22: variant/variant

† HWE = Hardy-Weinberg Equilibrium

Supplemental Table 3: other nominally statistically significant interactions between SNPs and dietary acrylamide intake

on the risk of estrogen receptor-positive breast cancer and clear differences in acrylamide dose-responses between genotypes, 20.3 years of follow-up

|  | Acrylamide, continuous intake |  | Acrylamide, tertiles of intake | | | | | | | | Interaction | |  |
| --- | --- | --- | --- | --- | --- | --- | --- | --- | --- | --- | --- | --- | --- |
| SNP | 10 µg/day |  | N cases | Tertile 1 | N cases | Tertile 2 | N cases | Tertile 3 |  | P for trend | P for linear interaction | |  |
|  |  |  |  |  |  |  |  |  |  |  | Raw p | Benjamini-Hochberg adjusted p value§ |  |
| Nominally statistically significant interactions | | | | | | | | | | | | |  |
| All |  |  |  |  |  |  |  |  |  |  |  |  |  |
| *NQO1*, rs1800566==0 | 0.99 (0.89-1.09) |  | 182 | Ref (1.00) | 192 | 1.06 (0.79-1.41) | 191 | 0.98 (0.73-1.31) |  | 0.87 | 0.04 | 0.42 |  |
| *NQO1*, rs1800566==1 | 0.87 (0.75-1.00) |  | 103 | Ref (1.00) | 94 | 0.96 (0.65-1.42) | 82 | 0.78 (0.52-1.17) |  | 0.23 |  |  |  |
| Never-smokers |  |  |  |  |  |  |  |  |  |  |  |  |  |
| *NQO1*, rs1800566==0 | 1.05 (0.93-1.19) |  | 95 | Ref (1.00) | 114 | 1.50 (1.00-2.24) | 115 | 1.26 (0.85-1.85) |  | 0.29 | 0.36 | 0.90 |  |
| *NQO1*, rs1800566==1 | 0.98 (0.80-1.20) |  | 58 | Ref (1.00) | 55 | 0.92 (0.54-1.56) | 49 | 0.96 (0.56-1.65) |  | 0.89 |  |  |  |
| All |  |  |  |  |  |  |  |  |  |  |  |  |  |
| *SLC7A11*, rs6838248==0 | 0.86 (0.73-1.02) |  | 60 | Ref (1.00) | 65 | 1.08 (0.61-1.89) | 62 | 0.86 (0.50-1.47) |  | 0.55 | 0.19 | 0.65 |  |
| *SLC7A11*, rs6838248==1 | 0.97 (0.89-1.07) |  | 226 | Ref (1.00) | 221 | 1.03 (0.80-1.34) | 211 | 0.95 (0.73-1.23) |  | 0.67 |  |  |  |
| Never-smokers |  |  |  |  |  |  |  |  |  |  |  |  |  |
| *SLC7A11*, rs6838248==0 | 0.79 (0.61-1.02) |  | 35 | Ref (1.00) | 34 | 0.80 (0.37-1.72) | 31 | 0.62 (0.31-1.24) |  | 0.18 | 0.04 | 0.44 |  |
| *SLC7A11*, rs6838248==1 | 1.08 (0.96-1.20) |  | 118 | Ref (1.00) | 135 | 1.41 (1.00-1.98) | 133 | 1.28 (0.91-1.80) |  | 0.17 |  |  |  |
| Clear differences in acrylamide dose-responses between genotypes | | | | | | | | | | | | | |
| All |  |  |  |  |  |  |  |  |  |  |  |  |  |
| *SOD1*, rs10432782=0 | 0.92 (0.84-1.01) |  | 220 | Ref (1.00) | 216 | 0.96 (0.74-1.25) | 211 | 0.88 (0.67-1.14) |  | 0.33 | 0.31 | 0.72 |  |
| *SOD1*, rs10432782==1 | 1.02 (0.87-1.20) |  | 66 | Ref (1.00) | 70 | 1.34 (0.81-2.21) | 62 | 1.10 (0.66-1.85) |  | 0.71 |  |  |  |
| Never-smokers |  |  |  |  |  |  |  |  |  |  |  |  |  |
| *SOD1*, rs10432782==0 | 0.99 (0.88-1.11) |  | 118 | Ref (1.00) | 123 | 1.13 (0.79-1.61) | 126 | 1.04 (0.74-1.46) |  | 0.87 | 0.08 | 0.49 |  |
| *SOD1*, rs10432782==1 | 1.23 (0.96-1.59) |  | 35 | Ref (1.00)) | 46 | 1.84 (0.90-3.77) | 38 | 1.45 (0.70-2.98) |  | 0.34 |  |  |  |
| All |  |  |  |  |  |  |  |  |  |  |  |  |  |
| *SOD2*, rs5746136==0 | 0.90 (0.79-1.01) |  | 154 | Ref (1.00) | 139 | 0.80 (0.58-1.11) | 124 | 0.69 (0.50-0.97) |  | 0.03 | 0.23 | 0.67 |  |
| *SOD2*, rs5746136==1 | 1.01 (0.90-1.13) |  | 132 | Ref (1.00) | 147 | 1.29 (0.92-1.80) | 149 | 1.22 (0.87-1.70) |  | 0.26 |  |  |  |
| Never-smokers |  |  |  |  |  |  |  |  |  |  |  |  |  |
| *SOD2*, rs5746136==0 | 0.96 (0.83-1.10) |  | 78 | Ref (1.00) | 84 | 1.13 (0.72-1.77) | 79 | 0.87 (0.56-1.35) |  | 0.49 | 0.09 | 0.49 |  |
| *SOD2*, rs5746136==1 | 1.18 (1.00-1.40) |  | 75 | Ref (1.00) | 85 | 1.35 (0.86-2.11) | 85 | 1.48 (0.96-2.31) |  | 0.08 |  |  |  |
| All |  |  |  |  |  |  |  |  |  |  |  |  |  |
| *SOD2*, rs4880==0 | 1.05 (0.90-1.23) |  | 59 | Ref (1.00) | 71 | 1.66 (1.02-2.73) | 69 | 1.42 (0.87-2.30) |  | 0.16 | 0.16 | 0.65 |  |
| *SOD2*, rs4880==1 | 0.91 (0.83-1.00) |  | 227 | Ref (1.00) | 215 | 0.88 (0.68-1.14) | 204 | 0.78 (0.60-1.01) |  | 0.06 |  |  |  |
| Never-smokers |  |  |  |  |  |  |  |  |  |  |  |  |  |
| *SOD2*, rs4880==0 | 1.15 (0.88-1.50) |  | 33 | Ref (1.00) | 46 | 2.05 (1.04-4.04) | 40 | 1.65 (0.84-3.23) |  | 0.13 | 0.43 | 0.90 |  |
| *SOD2*, rs4880==1 | 1.00 (0.89-1.12) |  | 120 | Ref (1.00) | 123 | 1.07 (0.75-1.52) | 124 | 0.98 (0.69-1.38) |  | 0.87 |  |  |  |
| All |  |  |  |  |  |  |  |  |  |  |  |  |  |
| *COMT*, rs737865==0 | 1.02 (0.92-1.13) |  | 143 | Ref (1.00) | 149 | 1.03 (0.75-1.41) | 152 | 1.10 (0.81-1.50) |  | 0.54 | 0.06 | 0.51 |  |
| *COMT*, rs737865==1 | 0.86 (0.76-0.97) |  | 143 | Ref (1.00) | 137 | 1.04 (0.74-1.47) | 121 | 0.72 (0.51-1.03) |  | 0.07 |  |  |  |
| Never-smokers |  |  |  |  |  |  |  |  |  |  |  |  |  |
| *COMT*, rs737865==0 | 1.12 (0.97-1.29) |  | 75 | Ref (1.00) | 91 | 1.33 (0.88-2.02) | 97 | 1.34 (0.91-1.99) |  | 0.18 | 0.05 | 0.44 |  |
| *COMT*, rs737865==1 | 0.93 (0.79-1.09) |  | 78 | Ref (1.00) | 78 | 1.15 (0.72-1.84) | 67 | 0.88 (0.55-1.43) |  | 0.52 |  |  |  |
| All |  |  |  |  |  |  |  |  |  |  |  |  |  |
| *SHBG*, rs6259==0 | 0.90 (0.82-0.99) |  | 229 | Ref (1.00) | 226 | 1.05 (0.81-1.36) | 204 | 0.79 (0.60-1.03) |  | 0.07 | 0.09 | 0.65 |  |
| *SHBG*, rs6259==1 | 1.13 (0.93-1.37) |  | 54 | Ref (1.00) | 58 | 1.06 (0.64-1.76) | 67 | 1.55 (0.92-2.62) |  | 0.10 |  |  |  |
| Never-smokers |  |  |  |  |  |  |  |  |  |  |  |  |  |
| *SHBG*, rs6259==0 | 0.97 (0.86-1.10) |  | 124 | Ref (1.00) | 135 | 1.29 (0.91-1.84) | 120 | 0.91 (0.64-1.30) |  | 0.57 | 0.06 | 0.44 |  |
| *SHBG*, rs6259==1 | 1.27 (0.98-1.65) |  | 29 | Ref (1.00) | 32 | 1.18 (0.56-2.50) | 42 | 2.19 (1.03-4.69) |  | 0.04 |  |  |  |
| All |  |  |  |  |  |  |  |  |  |  |  |  |  |
| HSD3*B1/B2*, rs1047303==0 | 0.88 (0.79-0.99) |  | 133 | Ref (1.00) | 142 | 1.05 (0.75-1.47) | 126 | 0.88 (0.62-1.23) |  | 0.43 | 0.15 | 0.65 |  |
| *HSD3B1/B2*, rs1047303==1 | 1.01 (0.90-1.13) |  | 153 | Ref (1.00) | 144 | 0.99 (0.71-1.36) | 147 | 0.95 (0.69-1.31) |  | 0.75 |  |  |  |
| Never-smokers |  |  |  |  |  |  |  |  |  |  |  |  |  |
| *HSD3B1/B2*, rs1047303==0 | 0.91 (0.77-1.07) |  | 64 | Ref (1.00) | 87 | 1.24 (0.79-1.97) | 69 | 0.97 (0.60-1.55) |  | 0.83 | 0.07 | 0.44 |  |
| *HSD3B1/B2*, rs1047303==1 | 1.13 (0.98-1.30) |  | 89 | Ref (1.00) | 82 | 1.31 (0.84-2.05) | 95 | 1.30 (0.86-1.97) |  | 0.22 |  |  |  |
| All |  |  |  |  |  |  |  |  |  |  |  |  |  |
| *UGT1A*, rs2070959==0 | 0.85 (0.75-0.97) |  | 151 | Ref (1.00) | 138 | 0.82 (0.58-1.15) | 124 | 0.70 (0.49-0.99) |  | 0.05 | 0.11 | 0.65 |  |
| *UGT1A*, rs2070959==1 | 1.03 (0.92-1.14) |  | 135 | Ref (1.00) | 148 | 1.18 (0.86-1.63) | 149 | 1.12 (0.81-1.54) |  | 0.51 |  |  |  |
| Never-smokers |  |  |  |  |  |  |  |  |  |  |  |  |  |
| *UGT1A*, rs2070959==0 | 0.92 (0.79-1.07) |  | 75 | Ref (1.00) | 89 | 1.02 (0.63-1.66) | 81 | 0.88 (0.55-1.40) |  | 0.55 | 0.10 | 0.49 |  |
| *UGT1A*, rs2070959==1 | 1.15 (0.98-1.34) |  | 78 | Ref (1.00) | 80 | 1.35 (0.87-2.08) | 83 | 1.32 (0.86-2.01) |  | 0.20 |  |  |  |
| All |  |  |  |  |  |  |  |  |  |  |  |  |  |
| *AKR1C1*, rs11252859==0 | 0.88 (0.77-1.01) |  | 119 | Ref (1.00) | 120 | 0.91 (0.63-1.32) | 91 | 0.71 (0.48-1.04) |  | 0.08 | 0.16 | 0.65 |  |
| *AKR1C1*, rs11252859==1 | 0.98 (0.89-1.09) |  | 167 | Ref (1.00) | 163 | 1.03 (0.76-1.38) | 181 | 1.04 (0.77-1.40) |  | 0.80 |  |  |  |
| Never-smokers |  |  |  |  |  |  |  |  |  |  |  |  |  |
| *AKR1C1*, rs11252859==0 | 1.06 (0.89-1.27) |  | 63 | Ref (1.00) | 66 | 1.14 (0.68-1.89) | 57 | 1.12 (0.67-1.86) |  | 0.66 | 0.89 | 0.97 |  |
| *AKR1C1*, rs11252859==1 | 1.02 (0.90-1.16) |  | 90 | Ref (1.00) | 101 | 1.28 (0.86-1.91) | 107 | 1.17 (0.79-1.74) |  | 0.46 |  |  |  |
| All |  |  |  |  |  |  |  |  |  |  |  |  |  |
| *AKR1C2*, rs11252887==0 | 1.00 (0.90-1.12) |  | 134 | Ref (1.00) | 142 | 1.07 (0.77-1.49) | 145 | 1.08 (0.78-1.52) |  | 0.64 | 0.19 | 0.65 |  |
| *AKR1C2*, rs11252887==1 | 0.89 (0.79-1.00) |  | 152 | Ref (1.00) | 139 | 0.92 (0.66-1.29) | 125 | 0.75 (0.53-1.05) |  | 0.10 |  |  |  |
| Never-smokers |  |  |  |  |  |  |  |  |  |  |  |  |  |
| *AKR1C2,* rs11252887==0 | 1.05 (0.92-1.20) |  | 74 | Ref (1.00) | 84 | 1.33 (0.85-2.08) | 87 | 1.36 (0.87-2.12) |  | 0.18 | 0.56 | 0.95 |  |
| *AKR1C2,* rs11252887==1 | 1.00 (0.85-1.18) |  | 79 | Ref (1.00) | 83 | 1.17 (0.74-1.84) | 75 | 0.95 (0.59-1.52) |  | 0.83 |  |  |  |
| All |  |  |  |  |  |  |  |  |  |  |  |  |  |
| *SULT1A1*, rs1042157==0 | 0.94 (0.82-1.07) |  | 96 | Ref (1.00) | 101 | 1.03 (0.70-1.52) | 106 | 1.01 (0.69-1.46) |  | 1.00 | 0.91 | 0.90 |  |
| *SULT1A1*, rs1042157==1 | 0.92 (0.83-1.02) |  | 189 | Ref (1.00) | 184 | 0.98 (0.73-1.31) | 163 | 0.79 (0.59-1.07) |  | 0.12 |  |  |  |
| Never-smokers |  |  |  |  |  |  |  |  |  |  |  |  |  |
| *SULT1A1*, rs1042157==0 | 1.08 (0.92-1.26) |  | 46 | Ref (1.00) | 63 | 1.58 (0.90-2.76) | 68 | 1.73 (1.01-2.96) |  | 0.05 | 0.83 | 0.97 |  |
| *SULT1A1*, rs1042157==1 | 0.99 (0.86-1.15) |  | 106 | Ref (1.00) | 106 | 1.06 (0.71-1.58) | 94 | 0.84 (0.57-1.26) |  | 0.39 |  |  |  |
| All |  |  |  |  |  |  |  |  |  |  |  |  |  |
| *SULT1A1*, rs6839==0 | 0.95 (0.85-1.06) |  | 115 | Ref (1.00) | 126 | 1.22 (0.87-1.73) | 121 | 1.05 (0.75-1.48) |  | 0.80 | 0.82 | 0.90 |  |
| *SULT1A1*, rs6839==1 | 0.91 (0.81-1.02) |  | 167 | Ref (1.00) | 157 | 0.87 (0.63-1.20) | 148 | 0.75 (0.54-1.04) |  | 0.08 |  |  |  |
| Never-smokers |  |  |  |  |  |  |  |  |  |  |  |  |  |
| *SULT1A1*, rs6839==0 | 1.05 (0.91-1.21) |  | 58 | Ref (1.00) | 76 | 1.69 (1.04-2.74) | 75 | 1.53 (0.95-2.45) |  | 0.08 | 0.92 | 0.97 |  |
| *SULT1A1*, rs6839==1 | 0.98 (0.84-1.15) |  | 94 | Ref (1.00) | 91 | 0.95 (0.61-1.47) | 87 | 0.82 (0.53-1.26) |  | 0.35 |  |  |  |
| All |  |  |  |  |  |  |  |  |  |  |  |  |  |
| *XPC*, rs2228001==0 | 1.04 (0.91-1.19) |  | 105 | Ref (1.00) | 105 | 0.97 (0.65-1.44) | 115 | 1.24 (0.83-1.87) |  | 0.28 | 0.32 | 0.72 |  |
| *XPC*, rs2228001==1 | 0.89 (0.80-0.99) |  | 181 | Ref (1.00) | 181 | 1.08 (0.81-1.45) | 158 | 0.78 (0.58-1.04) |  | 0.08 |  |  |  |
| Never-smokers |  |  |  |  |  |  |  |  |  |  |  |  |  |
| *XPC*, rs2228001==0 | 1.20 (0.98-1.46) |  | 58 | Ref (1.00) | 62 | 1.10 (0.63-1.94) | 72 | 1.77 (1.00-3.13) |  | 0.05 | 0.29 | 0.90 |  |
| *XPC*, rs2228001==1 | 0.97 (0.85-1.10) |  | 95 | Ref (1.00) | 107 | 1.42 (0.95-2.12) | 92 | 0.93 (0.64-1.36) |  | 0.65 |  |  |  |
| All |  |  |  |  |  |  |  |  |  |  |  |  |  |
| *MGC12965*, rs1280350==0 | 0.97 (0.89-1.06) |  | 198 | Ref (1.00) | 201 | 1.07 (0.81-1.41) | 193 | 1.04 (0.79-1.38) |  | 0.78 | 0.36 | 0.79 |  |
| *MGC12965*, rs1280350==1 | 0.86 (0.72-1.02) |  | 88 | Ref (1.00) | 85 | 0.96 (0.62-1.49) | 80 | 0.66 (0.42-1.02) |  | 0.06 |  |  |  |
| Never-smokers |  |  |  |  |  |  |  |  |  |  |  |  |  |
| *MGC12965*, rs1280350==0 | 1.07 (0.96-1.20) |  | 103 | Ref (1.00) | 120 | 1.44 (0.99-2.09) | 116 | 1.51 (1.04-2.19) |  | 0.03 | 0.16 | 0.69 |  |
| *MGC12965,* rs1280350==1 | 0.86 (0.66-1.12) |  | 50 | Ref (1.00) | 49 | 0.89 (0.48-1.63) | 48 | 0.50 (0.27-0.95) |  | 0.03 |  |  |  |
| All |  |  |  |  |  |  |  |  |  |  |  |  |  |
| *RRM2*, rs6759180==0 | 0.91 (0.81-1.02) |  | 148 | Ref (1.00) | 153 | 1.03 (0.74-1.42) | 119 | 0.83 (0.60-1.15) |  | 0.26 | 0.54 | 0.83 |  |
| *RRM2*, rs6759180==1 | 0.96 (0.86-1.09) |  | 134 | Ref (1.00) | 130 | 1.01 (0.72-1.40) | 150 | 0.95 (0.67-1.34) |  | 0.75 |  |  |  |
| Never-smokers |  |  |  |  |  |  |  |  |  |  |  |  |  |
| *RRM2*, rs6759180==0 | 0.93 (0.81-1.07) |  | 80 | Ref (1.00) | 93 | 1.34 (0.86-2.09) | 66 | 0.94 (0.60-1.47) |  | 0.80 | 0.05 | 0.44 |  |
| *RRM2*, rs6759180==1 | 1.19 (1.00-1.41) |  | 70 | Ref (1.00) | 74 | 1.20 (0.76-1.89) | 96 | 1.34 (0.86-2.08) |  | 0.20 |  |  |  |

§ proportion of false positives threshold set at 0.2

Hazard ratios are adjusted for age (years), age at menarche (years), age at menopause (years), age at first childbirth (nulliparous, 15-19 yrs, 20-24 yrs, 25-29 yrs, ≥30 yrs), parity (n children), ever use of oral contraceptives (yes/no), ever use of postmenopausal hormone treatment (yes/no), height (cm), body mass index (kg/m^2^), educational level (4 levels) energy intake (kcal/day), history of benign breast disease, family history of breast cancer, and in the analyses for all women: smoking status (never/ex/current smoker), smoking quantity (n cigarettes/day), smoking duration (smoking years)
